# Robust circulating microRNA signature for the diagnosis and early detection of pancreatobiliary cancer

**DOI:** 10.1186/s12916-025-03849-x

**Published:** 2025-01-21

**Authors:** Shuichi Mitsunaga, Masafumi Ikeda, Makoto Ueno, Satoshi Kobayashi, Masahiro Tsuda, Ikuya Miki, Takamichi Kuwahara, Kazuo Hara, Yukiko Takayama, Yutaro Matsunaga, Keiji Hanada, Akinori Shimizu, Hitoshi Yoshida, Tomohiro Nomoto, Kenji Takahashi, Hidetaka Iwamoto, Hideaki Iwama, Etsuro Hatano, Kohei Nakata, Masafumi Nakamura, Hiroko Sudo, Satoko Takizawa, Atsushi Ochiai

**Affiliations:** 1 Division of Biomarker Discovery, Exploratory Oncology Research and Clinical Trial Center, National Cancer Center, 6-5-1 Kashiwanoha, Kashiwa, Chiba 277-8577 Japan; 2 Department of Hepatobiliary and Pancreatic Oncology, National Cancer Center Hospital East, Kashiwa, Japan; 3 Department of Gastroenterology, Kanagawa Cancer Center, Yokohama, Japan; 4 Department of Gastroenterological Oncology, Hyogo Cancer Center, Akashi, Japan; 5 Department of Gastroenterology, Aichi Cancer Center Hospital, Nagoya, Japan; 6 Department of Internal Medicine, Institute of Gastroenterology, Tokyo Women’s Medical University Hospital, Tokyo, Japan; 7 Department of Surgery, Institute of Gastroenterology, Tokyo Women’s Medical University Hospital, Tokyo, Japan; 8 Department of Gastroenterology, Onomichi General Hospital, Onomichi, Hiroshima, Japan; 9 Department of Medicine, Division of Gastroenterology, Showa University School of Medicine, Tokyo, Japan; 10 Department of Medicine, Division of Gastroenterology, Asahikawa Medical University, Asahikawa, Japan; 11 Department of Gastroenterological Surgery, Hyogo College of Medicine, Nishinomiya, Japan; 12 Department of Surgery and Oncology, Graduate School of Medical Sciences, Kyushu University, Fukuoka, Japan; 13 Toray Industries, Inc, Kamakura, Japan; 14 Research Institute for Biomedical Sciences, Tokyo University of Science, Chiba, Japan

**Keywords:** Pancreatobiliary cancer, Circulating microRNA, Biomarker

## Abstract

**Background:**

A new circulating biomarker superior to carbohydrate antigen 19–9 (CA19-9) is needed for diagnosing pancreatobiliary cancer (PBca). The aim of this study was to identify serum microRNA (miRNA) signatures comprising reproducible and disease-related miRNAs.

**Methods:**

This multicenter study involved patients with treatment-naïve PBca and healthy participants. The optimized serum processing conditions were evaluated using *t*-distributed stochastic neighbor embedding (*t*-SNE) visualization. Serum miRNA candidates for disease association were selected using weighted gene coexpression network analysis (WGCNA). A miRNA signature combining multiple serum miRNAs was tested in exploratory, validation, and independent validation sets. The synthesis and secretion of diagnostic miRNAs were evaluated using human pancreatic cancer cells.

**Results:**

In total, 284 (150 healthy and 134 PBca) of 827 serum samples were processed within 2 h of blood collection before freezing, distributed in the same area as that in the *t*-SNE map, and assigned to an exploratory set. The 193 optimized samples were assigned to either the validation (50 healthy, 47 PBca) or independent validation (50 healthy, 46 PBca) set. Index-1, a combination of five serum miRNAs (hsa-miR-1343-5p, hsa-miR-4632-5p, hsa-miR-4665-5p, hsa-miR-665, and hsa-miR-6803-5p) with disease association in WGCNA, showed a sensitivity and specificity of > 80% and an AUC outperforming that of CA19-9 in the exploratory, validation, and independent validation sets. The AUC of Index-1 was superior to that of CA19-9 (0.856 vs. 0.649, *p* = 0.038) for detecting T1 tumors. miR-665, a component of Index-1, was expressed in human pancreatic cancer cells, and its transfection inhibited cell growth.

**Conclusions:**

The serum miRNA signature Index-1 is useful for detecting PBca and could facilitate the early diagnosis of PBca. These findings can help improve clinical PBca detection by providing an optimized biomarker that overcomes the limitations of the current standard.

**Supplementary Information:**

The online version contains supplementary material available at 10.1186/s12916-025-03849-x.

## Background

Pancreatobiliary cancer (PBca) is the leading cause of cancer-related deaths in Japan. The reported 5-year overall survival rate of patients with pancreatic and biliary tract cancer (BTC) in Japan was 0.8% and 24.5%, respectively, from 2009 to 2011 [1]. One of the reasons for the poor survival rate is the small percentage of resectable cases of pancreatic cancer (15–20%) [2] and BTC (40%) [3], as surgical resection is the only curative treatment for PBca. Blood tests are recommended as the first step for diagnosing PBca in clinical practice guidelines [4, 5]. The serum CA19-9 test is commonly used for detecting PBca in the clinic but is considered insufficient for detecting resectable cases. The diagnostic sensitivity of CA19-9 is reportedly 56–81% for pancreatic cancer overall [6–9], 40–47% for Union for International Cancer Control (UICC) stage I cancer, and 58–78% for UICC stage II cancer [10, 11]. In a BTC registry survey in Japan, elevated CA19-9 levels were recorded in only 69% of cases [5]. Therefore, new blood biomarkers are required for diagnosing PBca.

Circulating microRNA (c-miRNA) levels reflect the disease status of PBca. The expression of miR-21 increases in tumors during pancreatic carcinogenesis [6]. In addition, intratumoral miRNA expression in pancreatic cancer has been correlated with its expression in the blood [7]. In BTC, the increased expression of miRNAs such as miR-21 has been observed in the blood [8, 9]. c-miRNA signatures have shown sufficient accuracy for the diagnosis of pancreatic cancer [9, 12–16]. Hence, c-miRNA signatures may be useful biomarkers for detecting PBca, including resectable tumors. Our previous study revealed an overlap in serum miRNA expression profiles between pancreatic ductal carcinoma (PDAC) and BTC [9], suggesting the identification of common c-miRNA markers as an appropriate strategy for detecting both PDAC and BTC. Most miRNA genes within 50 kb of each other in the genome have been suggested to have highly correlated expression patterns [17]. Furthermore, miRNAs associated with the same disease tend to emerge as predefined groups [18]. In previous studies, when constructing miRNA-based discriminants, individual miRNAs had a low diagnostic ability for predicting physical deterioration compared with that of miRNA combinations [9, 19]. Hence, the combination of multiple disease-associated miRNAs appears to serve as a useful biomarker with high diagnostic ability. Weighted gene coexpression network analysis (WGCNA) has been widely applied for identifying candidate c-miRNA biomarkers in multiple diseases [20]. In PDAC, WGCNA has revealed four tumor miRNAs that are associated with pathological T factors, resulting in the construction of a prognostic signature consisting of these four tumor miRNAs [21]. Serum miRNA signatures constructed using multiple pancreatic- and BTC-related miRNAs through WGCNA appear to be promising PBca biomarkers with useful diagnostic performance.

Stability is a concern for the reproducible measurement of serum miRNA levels. Plasma and serum processing conditions can influence c-miRNA levels, as the amount and distribution of miRNAs differ greatly for various reasons, such as platelet contamination [22]. A principal component analysis using multiple facility samples revealed the degree of hemolysis as a facility-specific bias in plasma miRNA measurements [23]. Hemolysis during blood processing is typically caused by a delay between collection and separation and variation in centrifugation speed [24]. Hence, facility-specific bias was expected to be limited in the analysis of serum miRNA profiles from multiple facilities with unified and controlled processing conditions. The method of *t*-distributed stochastic neighbor embedding (*t*-SNE) is a state-of-the-art dimensionality reduction algorithm for nonlinear data representation; it creates a low-dimensional distribution or “map” of high-dimensional data (described in Additional File 1: Supplementary Methods). However, the utility of *t*-SNE for this purpose is yet to be confirmed. Therefore, this multicenter prospective study was aimed to describe differences in miRNA expression according to serum processing conditions using *t*-SNE visualization, identify disease-related miRNAs through WGCNA classification, and create diagnostic miRNA signatures for diagnosing PBca in exploratory, validation, and independent validation sets. We hypothesized that changes in serum miRNA profiles according to serum processing conditions could be visualized using *t*-SNE, thus enabling the selection of a reproducible serum miRNA profile. To the best of our knowledge, this is the first study to optimize clustering results using serum miRNA profiles according to serum processing conditions. Our approach allows for the identification of novel serum miRNA biomarkers with high specificity and sensitivity for the early clinical diagnosis of PBca.

## Methods

### Study design and participants

This prospective observational study was approved by the ethics review committee of the National Cancer Center Hospital East and National Cancer Center Hospital (approval no. 2016–049, 2020–449) and the institutional review boards of collaborating institutions. Patients who had treatment-naïve PBca and healthy volunteers aged > 60 years with no history of malignant disease or hospitalization in the past 3 months were registered for the study after providing written informed consent. The optimized serum processing conditions for miRNA measurements to determine robust and diagnostic serum miRNAs for creating a custom microarray and identifying serum miRNA signatures for PBca diagnosis were investigated using the exploratory set. The diagnostic value of serum miRNA signatures was confirmed using the validation and exploratory validation sets. A blood sample (6–10 mL) was obtained from each participant before the anticancer treatment.

A separate part of the study (study for time-course effects) recruited healthy volunteers from Toray Industries, Inc. (Kamakura, Japan) to investigate the time-course effects of serum processing conditions on c-miRNA levels. This study was approved by the Human Tissue Samples Ethics Committee for R&D of Toray Industries, Inc. (HC2017-10, HC2021-3, and HC2021-18).

All parts of the study complied with the principles of the Declaration of Helsinki and the Japanese Ethical Guidelines for Medical and Health Research Involving Human Subjects.

### Study for time-course effects

To investigate the time-course effects of storage on c-miRNA levels in whole blood, blood samples from the study for time-course effects were left at 23–27 °C (room temperature) for 30 min, 3 h, 6 h, or 9 h to coagulate before centrifugation at 2300 × *g* for 10 min at 25 °C. Serum samples were immediately collected and stored at − 80 °C. The time to centrifugation was determined as the room-temperature incubation time of whole blood.

To evaluate the time-course effects of storage on c-miRNA levels in sera, serum samples obtained from whole blood left for 30 min at 23–27 °C were allowed to stand for 30 min, 1 h, 2 h, 3 h, or 6 h at 23–27 °C and then frozen at − 80 °C. The time of serum processing was determined as the room-temperature incubation time of the serum.

### Stepwise serum processing of the exploratory set

Participants whose blood was collected from June 2016 to May 2017 were classified as Group 1. The time of blood processing was set at 6 h or less in Group 1. Participants who provided serum samples without any restrictions on serum processing from August 2016 to April 2018 were referred to as Group 2. The time of blood processing was 2 h or less in Groups 3, 4, and 5, whose sampling period was from March 2019 to July 2019, February 2019 to April 2020, and July 2019 to March 2020, respectively (Additional File 1: Supplementary Table S1). All serum samples were stored at − 80 °C until RNA extraction.

### Samples

Total RNA was extracted from 300 µL of serum using the 3D-Gene® RNA extraction reagent (Toray Industries, Inc., Kamakura, Japan) and then used for microarray analysis. The optimized serum preparation process is described in Additional File 1: Supplementary Methods.

The human pancreatic cancer cell lines KP-2 [JCRB0181], KP-4 [JCRB0182], SUIT-2 [JCRB1094], Capan-1 [ATCC: HTB-79], Miapaca-2 [ATCC: CRL-1420], CFPAC-1 [ATCC: CRL-1918], Panc-1 [ATCC: CRL-1469], and SW1990 [ATCC: CRL-2172] were purchased from the Japanese Collection of Research Bioresources Cell Bank (JCRB; Osaka, Japan) and the American Type Culture Collection (ATCC; Manassas, VA, USA). They were cultured according to the recommendations of the JCRB and ATCC. Total RNA from the cell lysate and culture supernatant was evaluated using microarray analysis. The proliferation of cells transfected with diagnostic miRNA mimics was evaluated as described in Additional File 1: Supplementary Methods.

### Microarray analysis

Two types of microarray were used in this study. Comprehensive miRNA expression analysis was performed using the 3D-Gene® Human microRNA Oligo Chip (Toray Industries, Inc.), which was designed to detect 2588 miRNAs registered in the miRBase database (release 21; https://www.mirbase.org/). This comprehensive microarray was tested using time-course and *t*-SNE analyses for the optimization of serum processing, selection of diagnostic miRNAs, and analysis of tissues, cells, and cell culture supernatants. A custom microarray was designed using the diagnostic miRNAs identified in this study and used to establish diagnostic miRNA signatures. Microarray experiments were conducted in accordance with the supplier’s instructions (Additional File 1: Supplementary Methods). A positive call for miRNA was defined as any microarray signal greater than the [mean + (2 × standard deviation)] of the negative control signals, from which the highest and least intense signals were removed. In the case of a missing value, the average value of all samples for the corresponding miRNA was employed. Each serum miRNA signal was normalized to that of the mean of three internal control miRNA signals, miR-149-3p, miR-2861, and miR-4463 (Int-con) [25].

### Serum processing optimization

The data of 25 previously identified PBca-related miRNAs [9] in Groups 1–5, described in “Stepwise serum processing of the exploratory set”, were included in the *t*-SNE analysis. The sample grouping of the 25 miRNAs was performed via *t*-SNE using Rtsne in R. Based on the results of the “*t*-SNE visualization of serum processing conditions” section below, we expected the processing conditions of Groups 3–5 to be appropriate. After *t*-SNE mapping of the populations in Groups 3–5 onto the same area visually showed the difference between Groups 1–2 and 3–5, the processing conditions of Groups 3–5 were determined to be optimal for serum processing. The details of the *t*-SNE analysis are provided in Additional File 1: Supplementary Methods.

### Statistical analyses for diagnostic miRNAs

The comprehensive miRNAs identified in the exploratory set with optimized serum processing were tested for robustness and disease association using WGCNA and for diagnostic probability using the ridge regression coefficient. The robustness of each miRNA was evaluated in terms of positive call rate and concentration linearity. We then examined whether the positive call rate for each miRNA was greater than 90% in the exploratory set. Concentration linearity was evaluated using a slope in linear regression and Pearson’s R-squared (rsq) between each normalized miRNA level and the Int-con signal strength, which was regarded as the serum miRNA concentration (data not shown). The difference in the slope between healthy volunteers in the exploratory set and that in the industrial data was calculated for each miRNA. A serum miRNA with rsq > 0.5 and slope difference < 0.1 was determined as a robust miRNA.

WGCNA was performed using normalized levels of the robust miRNAs from the exploratory set with optimized serum processing; it enabled the calculation of Pearson’s correlation coefficients between module eigengenes and clinical features, further providing disease-related modules for module–trait associations. Clinical features included tumor factors for PBca (healthy control: score 0, PBca patient: score 1), circulating levels of carcinoembryonic antigen (logCEA) and CA19-9 (logCA19-9), clinical T factor based on TNM classification eighth edition (healthy control: score 0; cT0/1: score 1; cT2: score 2; cT3: score 3; cT4: score 4), cN factor (healthy control: score 0; cN0: score 1; cN1: score 2), cM factor (healthy control: score 0; cM0: score 1; cM1: score 2), disturbance factors in serum miRNA measurement during the sample storage period at room temperature (within 120 min), and circulating platelet count. Of note, platelets are released during aggregation reactions, and platelet contamination affects the quantification of c-miRNAs [22, 26, 27]. Modules with a higher correlation with the disturbance factor were rejected. Disease-related miRNAs were identified as components of disease-related modules. Details of the WGCNA are described in Additional File 1: Supplementary Methods.

Ridge regression coefficients for linear discriminant analysis were used to select diagnostic miRNAs among disease-related miRNAs. In the best lambda using the cv.glmnet command in R’s glmnet package with default tenfold cross-validation, a ridge discriminant using all disease-related RNAs provided the coefficient of each disease-related miRNA. The miRNAs with the 16 largest coefficients were selected as diagnostic miRNAs.

### Statistical analyses for miRNA signature

Samples with optimized serum processing conditions among participants in the exploratory, validation, and independent validation sets were assigned to the respective sets. The levels of the 16 diagnostic miRNAs were determined for each sample. Fisher’s linear discriminant analysis was performed in the exploratory set with all combinations of five diagnostic miRNAs to create miRNA signatures.

**Fig. 1:**
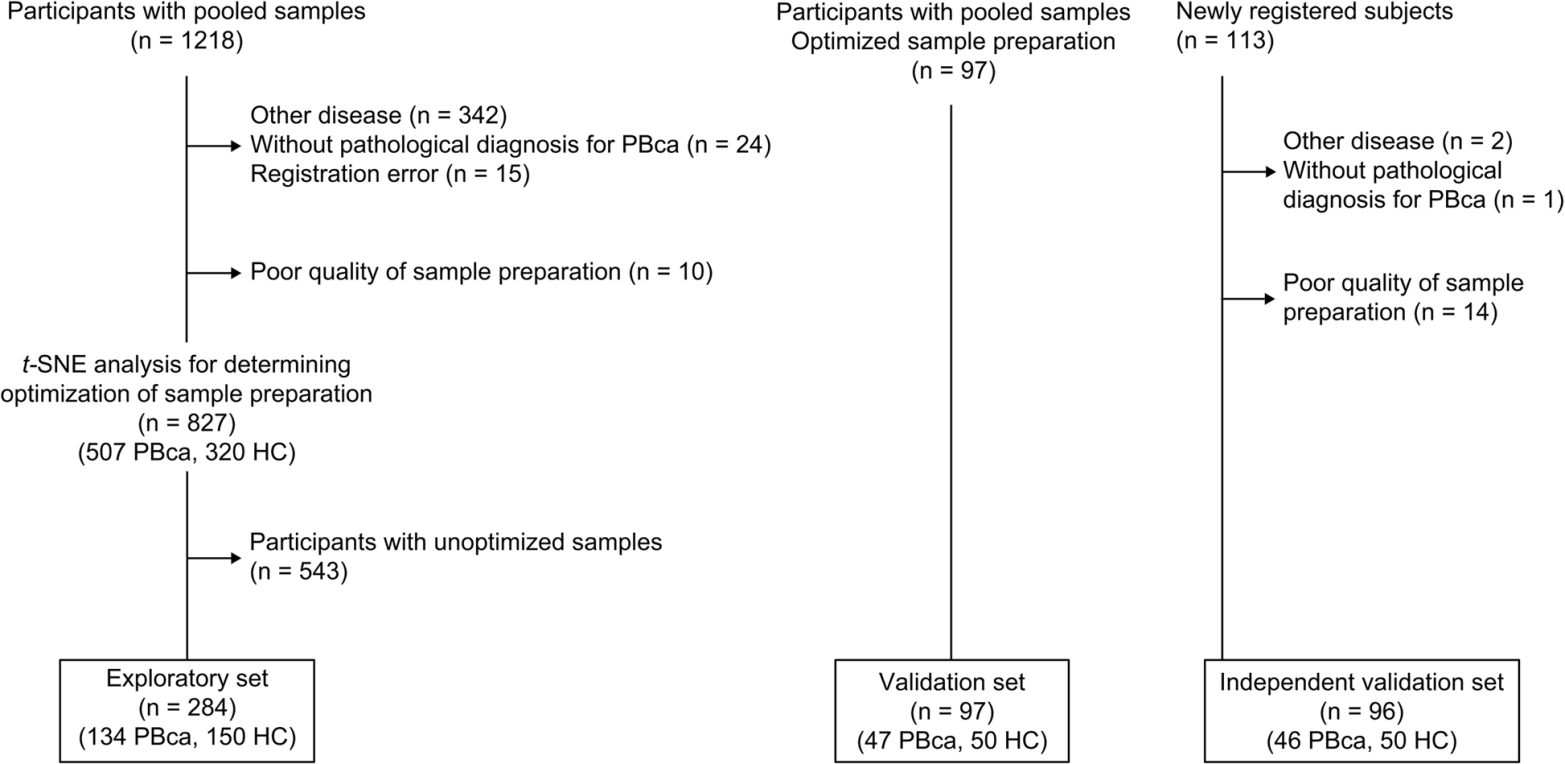
Clinical consort diagram. PBca pancreatic or biliary tract cancer, HC healthy control

To evaluate the diagnostic performance, sensitivity, and specificity of the miRNA signature, receiver operating characteristic (ROC) analysis was performed, and area under the curve (AUC) values were calculated using healthy volunteers as controls and patients with PBca as the test population. The sample size for the training set was determined using the steps shown in Fig. 1. The sample size of the validation and independent validation sets was set to 100 (healthy volunteers vs. patients with PBca, 1:1) for adequate discriminant analysis using five variables [28]. Samples in the exploratory set were divided into a 4:1 ratio and assigned to the four training groups and a validation group. This five-fold cross-validation was generated in 10 different ways, resulting in 50 groups. The c-miRNA signatures and CA19-9 levels were evaluated in these 50 groups, providing the mean sensitivity, mean specificity, and 95% confidence interval (CI) of AUC.

To determine the optimal number of variables, combinations of three to six miRNAs were employed to construct c-miRNA signatures in the exploratory set (Additional File 1: Supplementary Figure S1). Comprehensive combinations of the selected number of c-miRNAs were examined by the mean sensitivity, mean specificity, and 95%CI of the AUC. After determining the coefficients with specific variable miRNAs, we calculated the constants to maximize the separation between the groups, as well as the constants that resulted in 80% sensitivity or specificity, and obtained three kinds of discriminants for one c-miRNA signature. The performance improvement tended to plateau with combinations of five c-miRNAs.

The cutoff values for the c-miRNA signature and CA19-9 levels were set at 0 and 37 U/mL, respectively. Any miRNA signature that exceeded 80% sensitivity and 80% specificity and with a 95% CI for the AUC exceeding that of CA19-9 in the exploratory set was further evaluated in the validation and independent validation sets in the same way as that in the exploratory set. Two-group comparisons of numerical data were performed using an unpaired two-tailed Student’s *t*-test. The significance level was set at *p* < 0.05.

## Results

### Participants

A total of 1428 participants were enrolled in the study between June 2016 and April 2021 at 12 institutions in Japan and assigned to the exploratory set with stepwise serum processing (*n* = 827), the validation set (*n* = 97), or the independent validation set (*n* = 113) (Fig. 1). Consequently, we divided the participants with optimized serum processing into an exploratory set (*n* = 284), a validation set (*n* = 97), and an independent validation set (*n* = 96); their baseline characteristics are shown in Additional File 1: Supplementary Table S2. Similarly, 157 healthy volunteers selected from the volunteer resource of individuals aged ≥ 24 years were enrolled in the study for time-course effects. Among these, seven were assigned to a group for evaluating the time-course effects of storage at room temperature, whereas 150 were assigned to a group for evaluating the robustness of miRNAs.

### Time-course effects of storage at room temperature

**Fig. 2:**
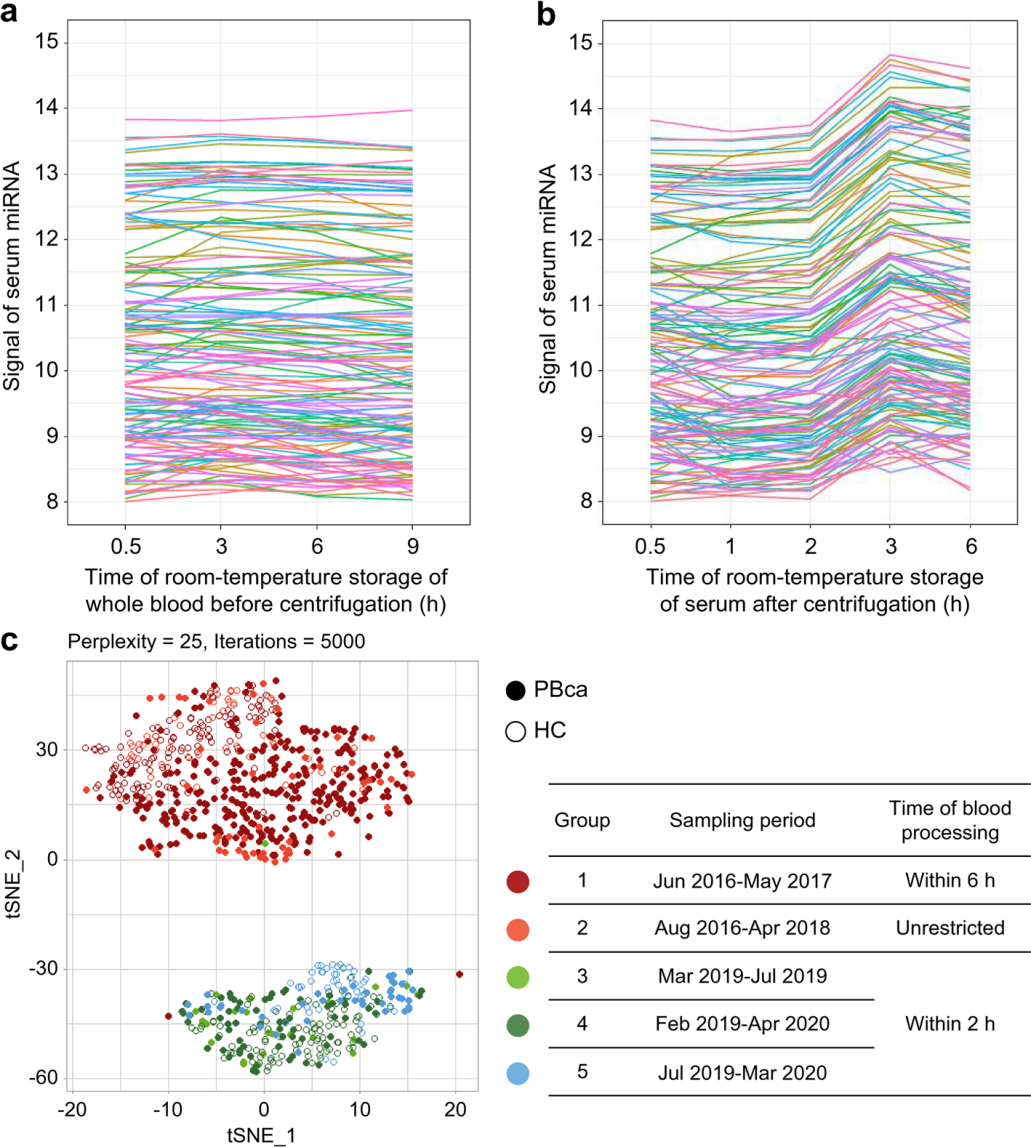
Optimal conditions for measuring serum miRNA levels. The kinetics of 147 miRNA signals in whole blood (**a**) and serum (**b**) are dependent on the time of serum preparation. The *x*-axis shows the time to centrifugation from blood collection (**a**) or time to freezing from the start of centrifugation after 30 min of coagulation (**b**). The *y*-axis shows the signal of each miRNA. **c** *t*-SNE plot of the 827 participants shown in Fig. 1, using a serum expression dataset of 25 PBca-related miRNAs. The 827 participants were grouped according to their sampling period. PBca, pancreatic or biliary tract cancer; HC, healthy control

We examined the effects of storage at room temperature using sample aliquots from seven healthy volunteers from the study for time-course effects. We observed that the time of room-temperature storage of whole blood (before centrifugation) did not significantly affect the serum levels of 721 miRNAs (average variation ± standard deviation [SD]: 0.02 ± 0.19 from 0.5 to 3 h; –0.03 ± 0.25 from 0.5 to 6 h) (Fig. 2a). In contrast, the storage of serum at room temperature for > 2 h affected the levels of miRNAs (average variation ± SD: –0.01 ± 0.30 from 0.5 to 1 h; 0.07 ± 0.38 from 0.5 to 2 h; 0.71 ± 0.43 from 0.5 to 3 h) (Fig. 2b). Hence, a time of blood processing ≤ 2 h was defined as the determinant of stable processing conditions for serum miRNA measurement.

### t-SNE visualization of serum processing conditions

Using 25 previously identified PBca-related miRNAs [9], we performed *t*-SNE analysis, which mapped 827 participants (320 healthy and 507 patients with PBca) into five groups (Groups 1–5) according to stepwise serum processing conditions (Fig. 2c). Groups 1 and 2 consisted of samples with storage times exceeding 2 h at room temperature and were distributed in the same area, separated from the plot distribution of Groups 3, 4, and 5, which contained sera with stable processing conditions. We also mapped 210 healthy participants from Groups 1, 4, and 5 in the* t*-SNE analysis with comprehensive miRNAs, confirming that the plot distribution of Group 1 was separated from that of Groups 4 and 5 (Additional File 1: Supplementary Figure S2).

The 284 samples from Groups 3, 4, and 5 (150 healthy participants and 134 patients with PBca) were assessed to show equivalent quality in the optimization and were assigned to the exploratory set for creating diagnostic miRNA signatures.

### Robust miRNAs

**Fig. 3:**
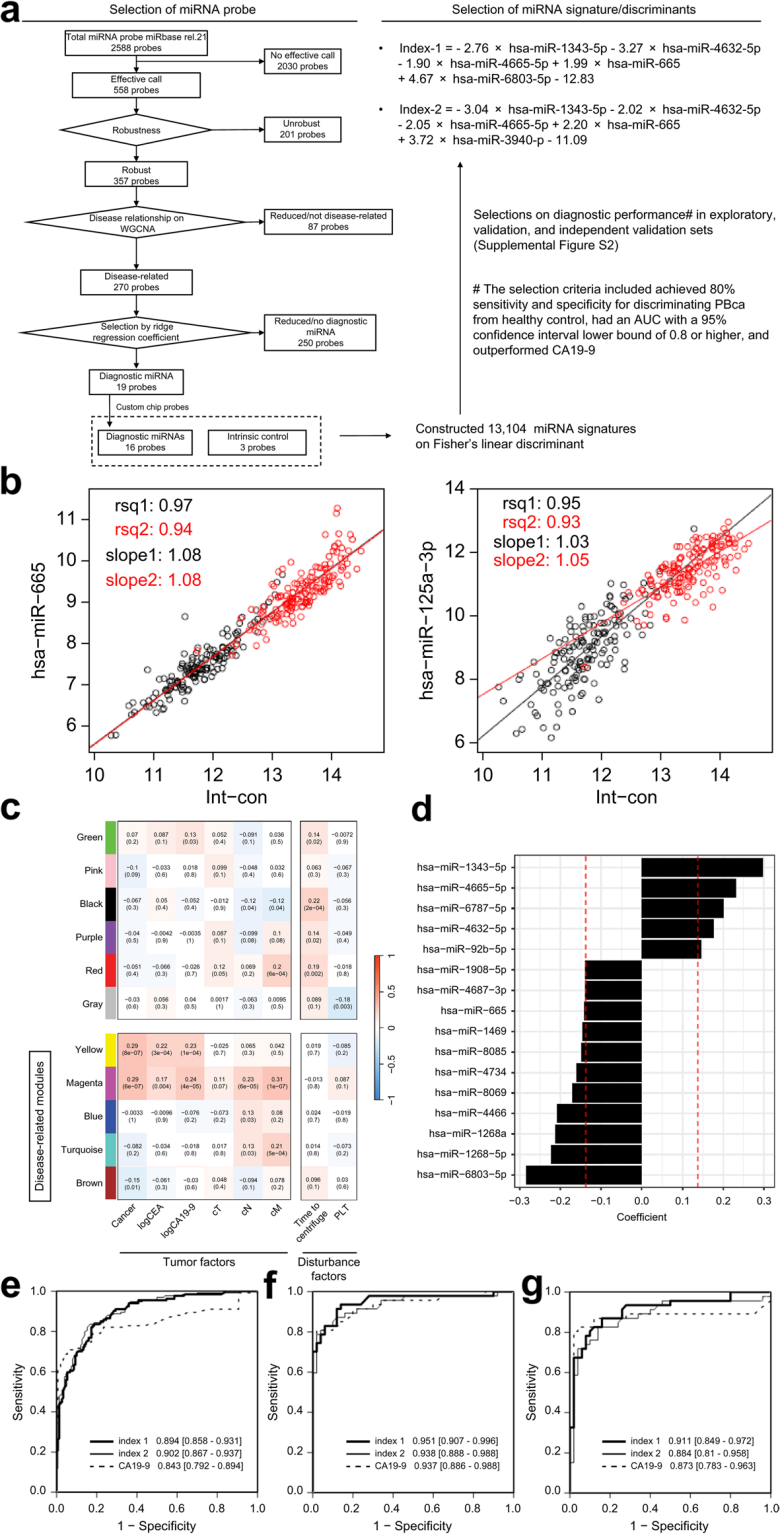
Selection of diagnostic miRNA candidates based on robustness and relationship with PBca. **a** Selection diagram of diagnostic miRNA candidates and miRNA signature for diagnostic index. **b** Examples of robustness evaluation of miRNAs. Left: example of robust miRNA, hsa-miR-665. Right: example of unrobust miRNA, hsa-miR-125a-3p. Evaluation was performed using two tests [black circle: HC in the exploratory set (set1, *n* = 150), red circle: HC in prior obtained data (set2, *n* = 150)]. *x*-axis: mean of three internal control miRNAs; *y*-axis: miRNA signals; rsq: regression squared of regression curve; slope: slope of regression curve. **c** WGCNA results: module–trait relationship between eigengene modules and clinical factors. Pearson correlation coefficients (*r*) and *p*-values are shown in each column. **d** Ridge regression coefficient for detecting PBCA in the exploratory set. *x*-axis: absolute values of ridge regression coefficients. Red dashed lines indicate the threshold for the selection of coefficients. ROC curves using Index-1, Index-2, or CA19-9 for discriminating patients with PBca from healthy participants in the exploratory (**e**), validation (**f**), and independent validation sets (**g**). AUC values are shown in each figure. Int-con, mean of internal control miRNAs; rsq, regression squared; slope, slope of regression line; PBca, pancreatic or biliary tract cancer; HC, healthy control; logCEA, CEA (U/mL) in logarithm with base 2; logCA19-9, CA19-9 (U/mL) in logarithm with base 2; cT, cN, cM: Union for UICC clinical TNM classification; PLT, platelet count

We found that among 2556 human miRNAs, 558 were detected as effective calls in the exploratory set (Fig. 3a; Additional File 1: Supplementary Table S3). We then used the Int-con signal to normalize miRNAs with positive calls in more than 99% of the samples from the exploratory set. The slope in the linear regression and Pearson’s rsq between the Int-con signal and each normalized miRNA level was calculated using the data of the 558 miRNAs from 150 healthy participants in the exploratory set or from 150 volunteers in the study for time-course effects (Fig. 3b). Based on the rsq status (> 0.5) and differences in slopes between the populations (< 0.1), we selected a total of 357 of the 558 miRNAs as robust miRNAs (Additional File 1: Supplementary Table S3).

### Disease-related miRNAs

We then performed hierarchical clustering of the 357 robust miRNA profiles from the exploratory set using WGCNA (a soft threshold of 5, a minimum module size of 10, and a deep split of 3) and obtained 11 miRNA modules (Additional File 1: Supplementary Figure S3a, b). The Pearson’s correlation coefficients between eigengene modules and each clinical feature are shown in Fig. 3c. The presence of PBca and serum levels of CEA and CA19-9 was associated with the yellow (*r* = 0.29, 0.22, and 0.23, respectively) and magenta (*r* = 0.29, 0.17, and 0.24, respectively) eigengene modules. We found a positive correlation between cN and the magenta (*r* = 0.23), blue (*r* = 0.13), and turquoise (*r* = 0.13) modules and between cM and the magenta (*r* = 0.31) and turquoise (*r* = 0.21) modules. The brown module was negatively correlated with the presence of PBca (*r* = –0.15). In contrast, the green, black, purple, and red modules were significantly correlated with sample storage time. We accordingly selected the yellow, magenta, blue, turquoise, and brown modules as the disease-related modules and analyzed 270 disease-related miRNAs in these modules as potential candidate biomarkers.

### Diagnostic miRNAs

We entered the 270 disease-related miRNA profiles from the exploratory set into a linear ridge regression model to create a discriminant for PBca, in which a ridge regression coefficient was assigned to each disease-related miRNA. The top 16 miRNAs in the profile of the 270 ridge regression coefficients were labeled as diagnostic miRNAs (Fig. 3d).

We considered these 16 diagnostic miRNAs as the most promising PBca discriminant markers and decided to create and evaluate a comprehensive linear discriminant model using all possible combinations of five arbitrarily chosen miRNAs. To construct the discriminant model, we created a custom microarray that included the above 16 marker candidates and the three internal control miRNAs.

### Establishment of the diagnostic miRNA signature Index-1

Using a custom microarray of diagnostic miRNAs, we analyzed a total of 284 serum samples from the exploratory set. In the linear discriminant analysis, we constructed 13,104 signatures using combinations of five chosen miRNAs (Fig. 3a). Among them, 136 signatures met the selection criteria: achieving 80% sensitivity and specificity for discriminating patients with PBca from healthy control, having an AUC with a 95% CI lower bound of 0.8 or higher, and outperforming CA19-9 (Additional File 1: Supplementary Figure S4). Index-1 and Index-2 of the 136 signatures met the criteria in both validation and independent validation sets; however, the remaining 134 signatures did not. The AUCs of Index-1 and Index-2 exceeded that of CA19-9 in the exploratory set (Index-1, 0.894; Index-2, 0.902; CA19-9, 0.834; Fig. 3e), validation set (Index-1, 0.951; Index-2, 0.938; CA19-9, 0.937; Fig. 3f), and independent validation set (Index-1, 0.911; Index-2, 0.884; CA19-9, 0.87; Fig. 3g). The formulas of Index-1 and Index-2 are shown in Fig. 3a.

The diagnostic performance of the combination of Index-1 and CA19-9 is shown in Additional File 1: Supplementary Table S4. If a positive sample was defined as either Index-1 > 0 or CA19-9 ≥ 37 U/mL, the overall sensitivity and specificity were 94.7% and 78.4%, respectively.

**Fig. 4:**
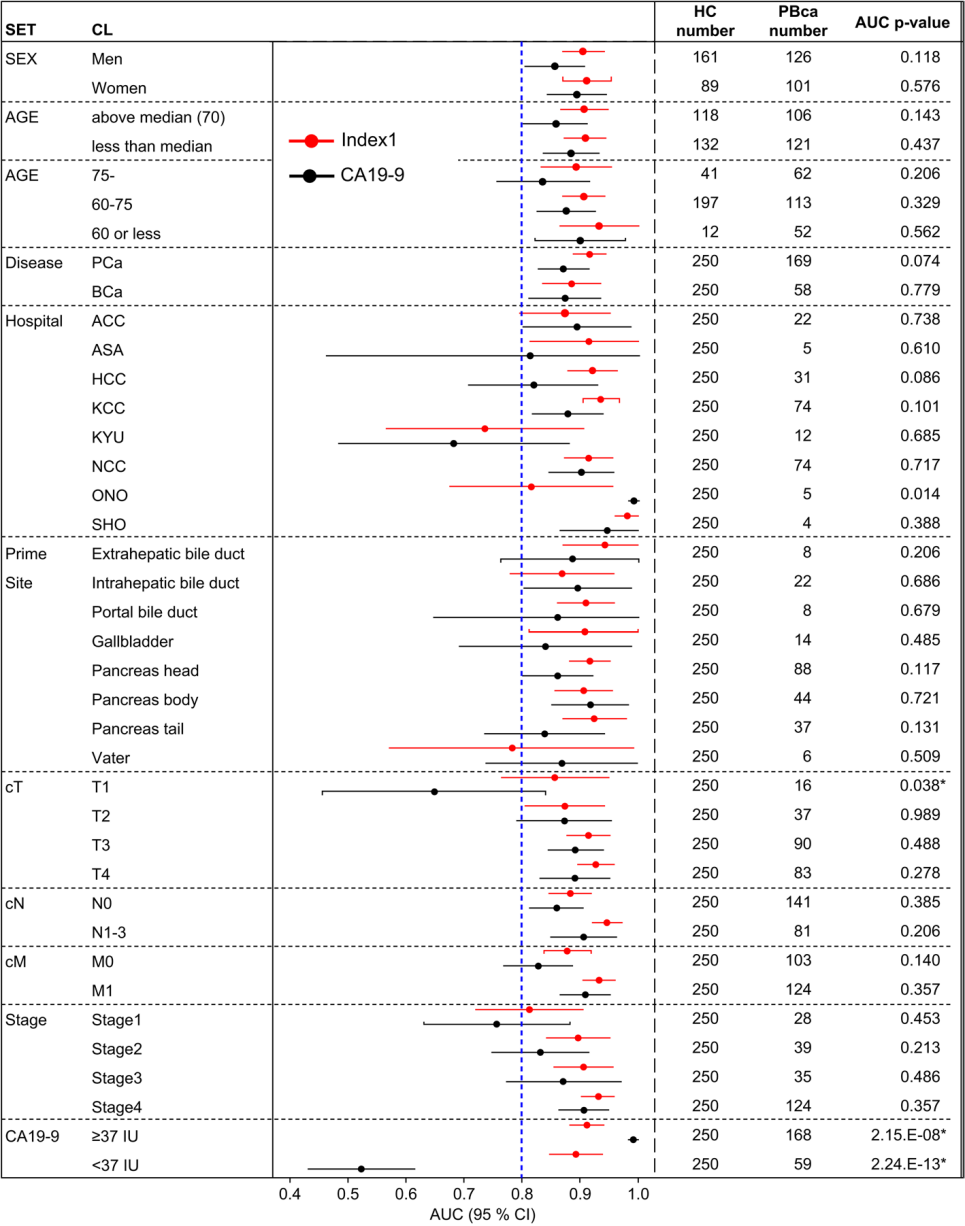
Subgroup analysis for diagnostic abilities of Index**-1.** The AUCs of Index-1 and CA19-9 in subgroups are shown horizontally. Circles represent the average AUC, and whiskers represent the lower and upper 95% confidence intervals. The red sign represents Index-1, whereas the black sign represents CA19-9. PBca, pancreatic or biliary tract cancer; HC, healthy control; AUC, area under the receiver operating characteristic curve; CI, confidence interval; CA19-9, carbohydrate antigen 19–9; ACC, Aichi Cancer Center; ASA, Asahikawa Medical University Hospital; HCC, Hyogo Cancer Center; KCC, Kanagawa Cancer Center; KYU, Kyusyu University Hospital; NCC, National Cancer Center East Hospital; ONO, JA Onomichi General Hospital; SHO, Showa University Hospital

The subgroup analysis results of Index-1 are shown in Fig. 4. The AUC of Index-1 for detecting T1 tumors (0.856, 95% CI [0.765–0.948]) was superior to that of CA19-9 (0.649, 95% CI [0.458–0.840], *p* = 0.038). The diagnostic ability of Index-1 for pancreatic cancer tended to be better than that of CA19-9 (AUC: 0.916 vs. 0.871, *p* = 0.074); however, no differences were observed between Index-1 and CA19-9 in their ability to detect BTC (AUC: 0.885 vs. 0.874, *p* = 0.779). In the population with CA19-9 levels < 37 or 15 U/mL [29], the sensitivity of Index-1 was 0.797 or 0.727, respectively (Additional File 1: Supplementary Table S5). The AUC of Index-1 in the population with CA19-9 levels < 37 U/mL was 0.896, whereas that in patients with CA19-9 level ≥ 37 U/mL was 0.887 (Fig. 4).

### Distributions of Index-1 and miRNA components

**Fig. 5:**
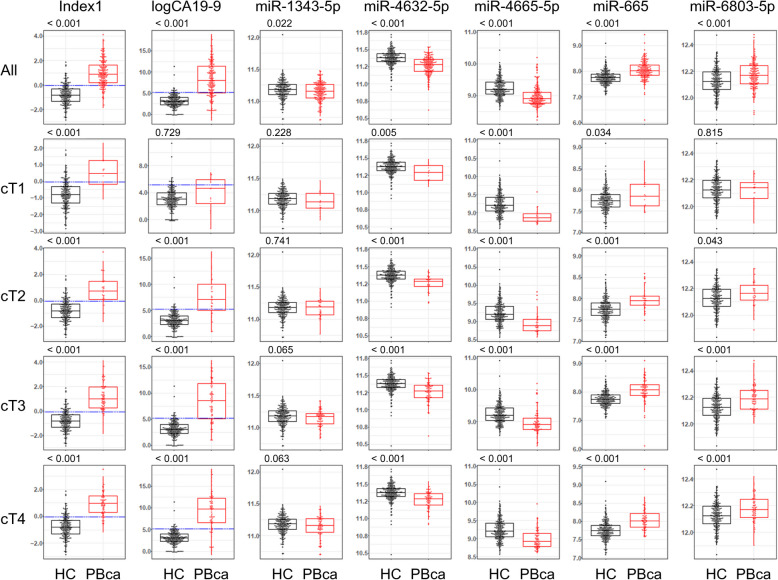
Index-1, CA19-9, and miRNAs of Index-1 in groups of cT. Healthy control (HC, black) and pancreatic/biliary tract cancer (PBCa, red) distributions are shown according to the UICC for International Cancer Control clinical T classification. Circle spots represent each case, boxes represent the interquartile range (IQR), and whiskers represent 1.5 × IQR from the hinge. The blue dotted lines in Index-1 and logCA19-9 indicate the thresholds. The *p*-value was calculated using Students’ *t*-test, either linearly (Index-1, CA19-9) or using log base of 2 (miRNAs), as shown in each graph. PBca, pancreatic or biliary tract cancer; HC, healthy control; logCA19-9: CA19-9 (U/mL) in logarithm with base 10. cT1 ~ cT4: UICC clinical T classification

In all of the exploratory, validation, and independent validation sets, the mean values of Index-1 and CA19-9 levels in patients with PBca were 0.97 and 11,068.85 U/mL, respectively, which were higher than those in healthy participants (Index-1: –0.78, *p* < 0.001; CA19-9: 25.28, *p* < 0.001) (Fig. 5). A weak correlation was found between Index-1 and CA19-9 levels (*r* = 0.127, *p* = 0.005). Among the five miRNAs of Index-1, miR-665 and miR-6803-5p showed elevated serum levels in patients with PBca compared with those in healthy participants (*p* < 0.001) (Fig. 5). The serum levels of the remaining three miRNAs were lower in patients with PBca than in healthy individuals: miR-1343-5p (*p* = 0.022), miR-4632-5p (*p* < 0.001), and miR-4665-5p (*p* < 0.001).

The distributions of Index-1, the five miRNAs, and CA19-9 according to cT status are shown in Fig. 5. We detected elevated levels of Index-1 (*p* < 0.001), miR-4632-5p (*p* = 0.005), miR-4665-5p (*p* < 0.001), and miR-665 (*p* = 0.034), but not CA19-9 (*p* = 0.729), in patients with PBca with T1 tumors compared with those in healthy participants.

### miR-665 in pancreatic cancer cell lines

**Fig. 6:**
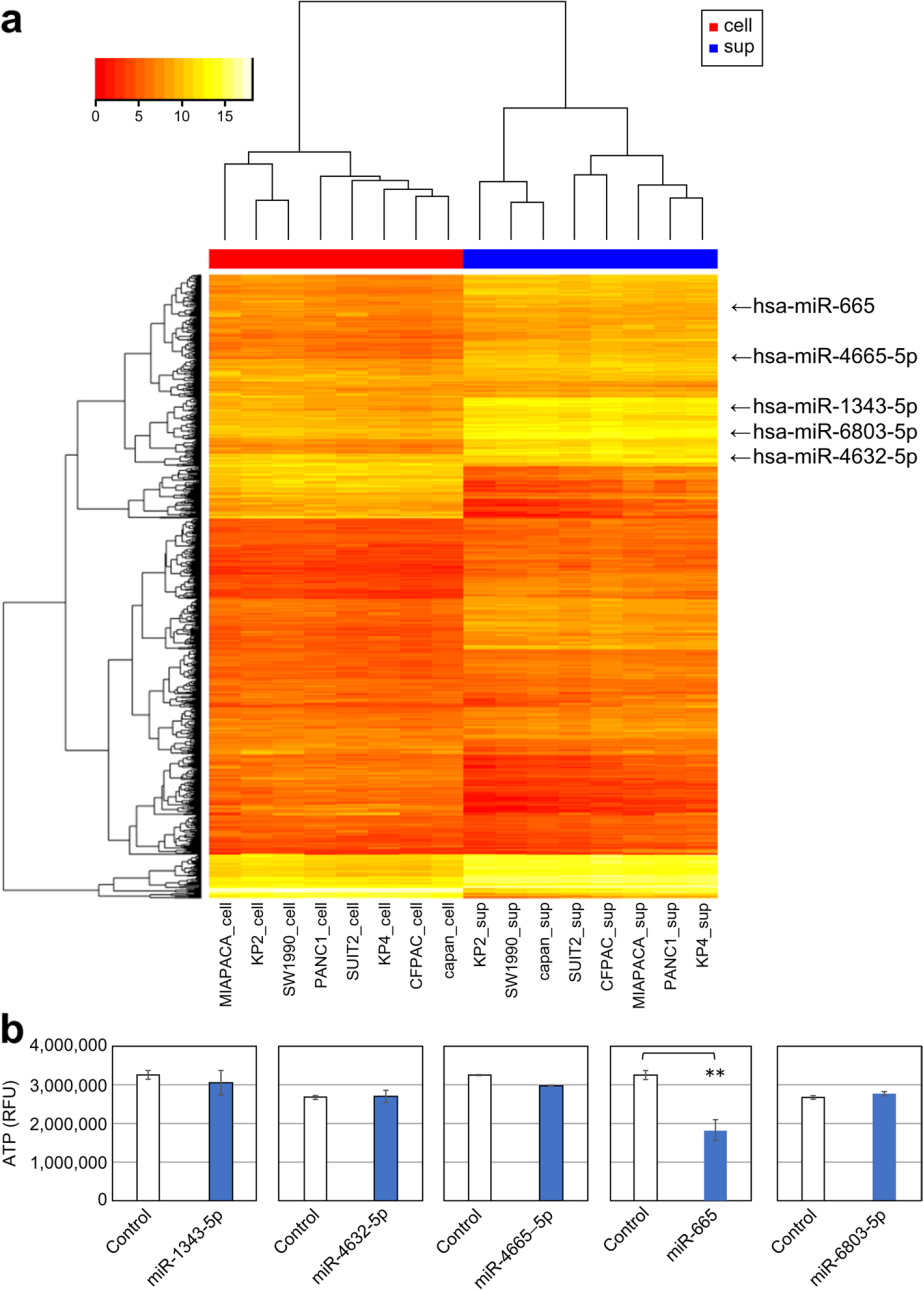
miRNA expression in pancreatic cancer lines and miR-665 effects on cell proliferation. **a** miRNA expression profile of eight cell lysate and supernatant samples. The upper color bar shows the origin tissue of each sample (red, cell lysate; blue, supernatant). Logarithmic miRNA expression is shown via color gradation. **b** Cell proliferation activity (ATP fluorescence) 4 days after induction with the miRNA mimic sequence. Data are presented as mean and SD, *n* = 3.**: *p* < 0.01, obtained via Student’s *t*-test. sup, supernatant; cell, cell lysate

We examined the profiles of comprehensive miRNAs, including the five miRNAs of Index-1, using supernatants and cell lysates from eight human pancreatic cancer cell lines (Fig. 6a). We observed that the five miRNAs showed relatively high expression in both cell lysates and supernatants.

Transfection with the miR-665 mimic inhibited the growth of Panc-1 cells on day 4 (*p* = 0.001) compared with that of negative control-transfected cells (Fig. 6b, Additional File 1: Supplementary Table S6). In contrast, we did not observe any cell growth inhibition following transfection with the other four miRNA mimics.

## Discussion

The PBca diagnostic performance of the serum miRNA signature Index-1 competed with that of the established serum biomarker CA19-9 [4, 5]. In this study, the sensitivity and specificity threshold for acceptable performance for PBca diagnosis was set at ≥ 80% based on the reported diagnostic abilities of CA19-9 [6–9]. Index-1 showed acceptable diagnostic performance in the exploratory, validation, and independent validation sets. The subpopulation analysis revealed that compared with CA19-9, Index-1 had superior diagnostic ability in patients with PBca with UICC T1 staging and in those with pancreatic cancer (Fig. 4), which is important for detecting cases of resectable pancreatic cancer. T1-staged tumors have a better prognosis in resected pancreatic cancer, independent of the status of lymph node metastasis [30]. Therefore, T1 tumor detection using Index-1 has the potential to improve the therapeutic results in patients with pancreatic cancer. In addition, Index-1 showed high accuracy in the patient group with CA19-9 levels < 37 or 15 U/mL (Additional File 1: Supplementary Table S5). Patients with gastrointestinal cancer with the Lewis (a- b-) phenotype (Lewis-negative) cannot synthesize the sialyl Lewis A antigen CA19-9 [31]. Dupan-2, a serum biomarker for pancreatic cancer diagnosis, was elevated in 5 of 13 patients with Lewis-negative pancreatic cancer [29]. The sensitivity of Index-1 for detecting PBca and pancreatic cancer was 79.7% and 82.5% in the population with CA19-9 levels < 37 U/mL and 72.7% and 69.6% in the population with CA19-9 levels < 15 U/mL, respectively (Additional File 1: Supplementary Table S5). The diagnostic ability of Index-1 appears to be superior to that of Dupan-2 for Lewis-negative pancreatic cancer. Lewis-negative tumors were found in 30% of patients with pancreatic cancer [29], in whom Index-1 would show appropriate diagnostic ability. Furthermore, the combined performance of Index-1 and CA19-9 appeared to have higher sensitivity for detecting PBca than that of CA19-9 or Index-1 alone in the exploratory (92.5% vs. 69.4% or 82.1%), validation (97.9% vs. 80.9% or 91.5%), and independent validation (97.8% vs. 80.4% or 87.0%) sets (Additional File 1: Supplementary Table S4). These results indicate that the serum miRNA signature Index-1 is a useful biomarker for PBca diagnosis.

The degree of tumor extension was evaluated using T-staging (UICC 8th edition [32]). The CA19-9 levels in patients with PBca gradually increased according to the degree of T-staging. The differences in the CA19-9 levels between healthy volunteers and patients with T1 tumors were small compared with those between healthy volunteers and patients with T2/3/4 tumors. However, the serum levels of Index-1 in the PBca population with T1 tumors were higher than those in healthy volunteers (Fig. 5). Index-1 was calculated using the following formula: Index-1 = –2.76 × hsa-miR-1343-5p – 3.27 × hsa-miR-4632-5p – 1.90 × hsa-miR-4665-5p + 1.99 × hsa-miR-665 + 4.67 × hsa-miR-6803-5p – 12.83 (Fig. 3a). An elevation in Index-1 levels was dependent on increasing miR-665 and miR-6803-5p levels and decreasing miR-1343-5p, miR-4632-5p, and miR-4665-5p levels. The elevation in serum Index-1 levels in patients with PBca with T1 tumors can be attributed to a decrease in serum miR-4665-5p level compared with that in healthy volunteers (Fig. 5). Such changes could be useful for detecting T1 tumors in PBca.

As PBca diagnosis via liquid biopsy is a popular research area, many researchers have focused on miRNAs. In addition to our previous study [9], recently, Huang et al. (hsa-miR-132-5p, hsa-miR-30c-5p, hsa-miR-24-3p, and hsa-miR-23a-3p) [33], Huang et al. (hsa-miR-4486, and hsa-miR-6075) [34], and Shi et al. (hsa-miR-1246, hsa-miR-205-5p, and hsa-miR-191-5p) [35] have reported on the diagnostic performance of c-miRNAs in pancreatic cancer. These c-miRNA markers were also tested in this study; however, we could not detect any of them. This discrepancy in the findings might be attributed to differences in methodology or sample quality.

The incubation time from serum separation to freezing (time of serum processing) affected the serum miRNA expression profiles in this study. Most serum miRNA expression levels changed after 2 h of storage at room temperature (Fig. 2b). However, the serum miRNA levels were not affected after the storage of whole blood at room temperature, even 9 h after blood collection (Fig. 2a). The fragility of serum miRNAs could be attributed to ribonuclease activity, which is higher in serum than in whole blood, possibly due to the release of ribonuclease from platelets [36]. Mapping using *t*-SNE analysis of the 25 serum miRNA expression profiles in the 507 patients with PBca and 327 healthy volunteers comprised two groups classified according to the time of blood processing, that is, within 2 h or more (Fig. 2c). Furthermore, clusters formed due to the time of serum processing in 210 healthy participants in the analysis of serum processing optimization (Additional File 1: Supplementary Figure S2). The comprehensive miRNA analysis using healthy participants also supported these clusters. Our findings therefore characterized the time of serum processing as a disturbance factor in the reproducible measurement of serum miRNA levels, as well as platelet contamination [22], though some disturbance factors remained unknown. These results suggest that facility-specific bias can be reduced by limiting processing time and platelet contamination, potentially improving the feasibility of inter-facility comparisons, although further studies are needed to reveal additional factors.

One of the five serum miRNAs of Index-1, miR-665, was confirmed to be present in various pancreatic cancer cells and their culture media in vitro (Fig. 6a). Accordingly, the administration of the intracellular miR-665 mimic inhibited the proliferation of Panc-1 cells (Fig. 6b). miR-665 has been reported to suppress tumor growth in not only pancreatic cancer [37] but also other types of cancer [38–44]. Intracellular miR-665 transfection led to the decreased expression of *TGFBR1* and *TGFBR2*, suppressing the proliferation and invasion of human pancreatic cancer cells [37]. The tumor-suppressive roles of miR-665 have also been reported in gastric cancer [38–40], ovarian cancer [41, 42], and hepatocellular carcinoma [43, 44]. However, tumor-suppressive miRNAs appear to be preferentially discharged from cancer cells [45, 46]. Mizoguchi et al. suggested that tumor-suppressive miR-8073 enclosed in the form of extracellular vesicles was more actively discharged from colorectal cancer cells than from normal cells [45]. Similarly, Kanlikilicer et al. reported that tumor-suppressive miR-6126 was ubiquitously released in high abundance from ovarian cancer cells via extracellular vesicles [46]. Circulating miR-665 may be secreted from tumor cells to facilitate their proliferation. However, further research is needed to understand the detailed function of miR-665 in pancreatic cancer.

A limitation of this study was the imputation of missing values. We used the average value of the other samples for missing data imputation, which may have resulted in a model misspecification bias. To reduce imputation-related effects, serum miRNAs with high effective call rates (> 90%) were selected as marker candidates for testing robustness. The effective call rate for the five miRNAs in Index-1 was 100% in the exploratory set. Therefore, the diagnostic value of Index-1 limited the effects of imputation-related bias. Another limitation is the considerable variation in the number of participants provided by each institution (ranging from fewer than 5 to 74 cases, Additional File 1: Supplementary Table S2). This variation, especially when observed between the exploratory, validation, and independent validation sets, could potentially introduce bias into the model construction. A higher predominance of early stages was found in the exploratory set compared to that in the validation and independent validation sets. A concern was the potential loss of detection ability for advanced stages in a diagnostic biomarker from the exploratory set. To address our concerns, diagnostic miRNAs were selected from disease-related miRNAs which exhibited a positive correlation with TNM classification and circulating CA19-9 levels. Subsequently, miRNA signatures were created using multiple diagnostic miRNAs. Diagnostic miRNA levels were expected to be high in the population of individuals in advanced stages as opposed to those in early stages, thereby enhancing the efficacy of the miRNA signature in diagnosing advanced stages. The findings indicate that Index-1 demonstrated a higher diagnostic value compared to CA19-9 in both early and advanced stages, as revealed by the subpopulation analysis of all three datasets (Fig. 4). Therefore, we believe that the impact of this discrepancy is minimal. However, further validation using additional datasets is needed to apply our findings in clinical practice.

In addition, several challenges remain regarding the use of this test in clinical settings. For example, microarray analysis may have limitations when evaluated using other platforms. Although we have developed a cost-effective custom microarray, opening up possibilities for large-scale testing, the implementation of alternative methods such as reverse transcription-quantitative polymerase chain reaction (RT-qPCR) may also be considered. However, evaluating Index-1 using RT-qPCR or next-generation sequencing may introduce bias in the measurement of each miRNA level owing to the amplification steps in these methods, resulting in the modification of the discriminant formula.

## Conclusions

A serum miRNA signature, Index-1, was established as a useful biomarker for PBca diagnosis. The diagnostic ability of Index-1 was retained in populations with low CA19-9 levels or T1 tumors, hence improving the early diagnosis of PBca. Our study offers novel insights into the use of serum miRNAs as biomarkers for PBca diagnosis and provides a basis for improving facility-specific bias through the regulation of appropriate sample processing conditions. Further studies are required to elucidate the mechanism of action of the five Index-1 miRNAs in the context of cancer association and extracellular release.

## Supplementary Information

Additional file 1: Supplemental Material: Robust circulating microRNA signature for the diagnosis and early detection of pancreatobiliary cancer. Supplementary Methods. Supplementary References. Figure S1. Performance of discriminants with three to six miRNA variables. Figure S2. *t*-SNE plot of comprehensive miRNAs in healthy control serum. Figure S3. WGCNA optimization of 357 robust miRNAs. Figure S4. Performance of 136 miRNA discriminants. Table S1. Time until sample freezing. Table S2. Clinical background. Table S3. Features of microRNA probes. Table S4. Discriminatory performance of Index-1, CA19-9, and their combination. Table S5. Discriminatory performance of Index-1 when changing the threshold for CA19-9 levels. Table S6. miRNA expression in pancreatic cancer lines and miR-665 effects on cell proliferation.

## Data Availability

The microarray data obtained in this study complied with the Minimum Information About a Microarray Experiment guidelines and are publicly available through the Gene Expression Omnibus (GEO, https://www.ncbi.nlm.nih.gov/geo/query/acc.cgi?acc=GSE268524). Requests for data and samples should be made to the corresponding author, Shuichi Mitsunaga, via email at smitsuna@east.ncc.go.jp. Data and samples will be made available upon the approval of reasonable requests to the lead author (Shuichi Mitsunaga) and Toray Industries, Inc.

## Abbreviations

ATCC: American Type Culture Collection
AUC: Area under the curve
BTC: Biliary tract cancer
CA19-9: Carbohydrate antigen 19–9
logCA19-9: Circulating level of CA19-9
logCEA: Circulating level of CEA
c-miRNA: Circulating miRNA
CI: Confidence interval
Int-con: Internal control
JCRB: Japanese Collection of Research Bioresources Cell Bank
miRNA: MicroRNA
PDAC: Pancreatic ductal carcinoma
PBca: Pancreatobiliary cancer
RT-qPCR: Reverse transcription quantitative polymerase chain reaction
ROC: Receiver operating characteristic
Rsq: R-squared
SD: Standard deviation
*t*-SNE: *t*-Distributed stochastic neighbor embedding
UICC: Union for International Cancer Control
WGCNA: Weighted gene coexpression network analysis
