# Correction: Robust circulating microRNA signature for the diagnosis and early detection of pancreatobiliary cancer

**DOI:** 10.1186/s12916-025-04079-x

**Published:** 2025-04-16

**Authors:** Shuichi Mitsunaga, Masafumi Ikeda, Makoto Ueno, Satoshi Kobayashi, Masahiro Tsuda, Ikuya Miki, Takamichi Kuwahara, Kazuo Hara, Yukiko Takayama, Yutaro Matsunaga, Keiji Hanada, Akinori Shimizu, Hitoshi Yoshida, Tomohiro Nomoto, Kenji Takahashi, Hidetaka Iwamoto, Hideaki Iwama, Etsuro Hatano, Kohei Nakata, Masafumi Nakamura, Hiroko Sudo, Satoko Takizawa, Atsushi Ochiai

**Affiliations:** 1 Division of Biomarker Discovery, Exploratory Oncology Research and Clinical Trial Center, National Cancer Center, 6-5-1 Kashiwanoha, Kashiwa, Chiba 277-8577 Japan; 2 Department of Hepatobiliary and Pancreatic Oncology, National Cancer Center Hospital East, Kashiwa, Japan; 3 Department of Gastroenterology, Kanagawa Cancer Center, Yokohama, Japan; 4 Department of Gastroenterological Oncology, Hyogo Cancer Center, Akashi, Japan; 5 Department of Gastroenterology, Aichi Cancer Center Hospital, Nagoya, Japan; 6 Department of Internal Medicine, Institute of Gastroenterology, Tokyo Women’s Medical University Hospital, Tokyo, Japan; 7 Department of Surgery, Institute of Gastroenterology, Tokyo Women’s Medical University Hospital, Tokyo, Japan; 8 Department of Gastroenterology, Onomichi General Hospital, Onomichi, Hiroshima, Japan; 9 Department of Medicine, Division of Gastroenterology, Showa University School of Medicine, Tokyo, Japan; 10 Department of Medicine, Division of Gastroenterology, Asahikawa Medical University, Asahikawa, Japan; 11 Department of Gastroenterological Surgery, Hyogo College of Medicine, Nishinomiya, Japan; 12 Department of Surgery and Oncology, Graduate School of Medical Sciences, Kyushu University, Fukuoka, Japan; 13 Toray Industries, Inc, Kamakura, Japan; 14 Research Institute for Biomedical Sciences, Tokyo University of Science, Chiba, Japan

**Correction: BMC Med 23, 23 (2025)**

**https://doi.org/10.1186/s12916-025-03849-x**

The original manuscript has been updated with an amendment to a minor typo in co-author, Satoshi Kobayashi’s name [1].
