# Supplementary material for: Robust circulating microRNA signature for the diagnosis and early detection of pancreatobiliary cancer
Source: BMC Med. 2025 Jan 21;23:23. doi: 10.1186/s12916-025-03849-x (PMC11752661; doi:10.1186/s12916-025-03849-x)
Supplement: Supplementary file 1 — Additional file 1: Supplemental Material: Robust circulating microRNA signature for the diagnosis and early detection of pancreatobiliary cancer. Supplementary Methods. Supplementary References. Figure S1. Performance of discriminants with three to six miRNA variables. Figure S2. t-SNE plot of comprehensive miRNAs in healthy control serum. Figure S3. WGCNA optimization of 357 robust miRNAs. Figure S4. Performance of 136 miRNA discriminants. Table S1. Time until sample freezing. Table S2. Clinical background. Table S3. Features of microRNA probes. Table S4. Discriminatory performance of Index-1, CA19-9, and their combination. Table S5. Discriminatory performance of Index-1 when changing the threshold for CA19-9 levels. Table S6. miRNA expression in pancreatic cancer lines and miR-665 effects on cell proliferation. [file 12916_2025_3849_MOESM1_ESM.docx]

**Additional File 1**

**Supplemental Material: Robust circulating microRNA signature for the diagnosis and early detection of pancreatobiliary cancer**

In this file:

**Supplementary Methods**

**Supplementary References**

Supplementary Figures S1–S4

**Figure S1**. Performance of discriminants with three to six miRNA variables.

**Figure S2**. *t*-SNE plot of comprehensive miRNAs in healthy control serum.

**Figure S3**. WGCNA optimization of 357 robust miRNAs.

**Figure S4**. Performance of 136 miRNA discriminants.

Supplementary Tables S1–S6

**Table S1**. Time until sample freezing.

**Table S2**. Clinical background.

**Table S3**. Features of microRNA probes.

**Table S4**. Discriminatory performance of Index-1, CA19-9, and their combination.

**Table S5**. Discriminatory performance of Index-1 when changing the threshold for CA19-9 levels.

**Table S6**. miRNA expression in pancreatic cancer lines and miR-665 effects on cell proliferation.

**Supplementary Methods**

***Optimized serum processing in validation and independent validation cohorts***

Blood samples were left at 23–27°C (room temperature) for up to 1 h for coagulation before being centrifuged at 2300 × *g* and 25°C for 10 min. The serum was collected within 1 h of the start of centrifugation and stored at –80°C until RNA extraction. The blood collection tubes were standardized to VP-AS 106KM60 (Terumo, Tokyo, Japan), the same as in the exploratory set.

***Microarray analysis***

Half of the extracted total RNA from 300 µL serum was used for comprehensive miRNA analysis. For the analysis of PBca marker candidates using custom microarrays, one-eighth of the serum-extracted RNA was labeled using the 3D-Gene^®^ microRNA Labelling Kit (Toray Industries, Inc., Kamakura, Japan). All miRNA analyses were performed in accordance with the supplier's instructions. A positive call for miRNA was defined as a microarray signal greater than the [mean + (2 × standard deviation)] of negative control signals, of which the highest and least intense signals were removed and log-transformed on a base-2 logarithm scale. Serum miRNA signals across different microarrays were normalized using the internal control correction coefficient (Int-con). The mean of three internal control miRNA signals (miR-149-3p, miR-2861, and miR-4463) served as the Int-con [1]. Each data point was normalized based on the value obtained by subtracting the Int-con from 12.6 (average Int-con value, house data). Each data point was then multiplied by the miRNA-specific adjustment coefficient previously obtained from the miRNA-specific calibration curve. Accordingly, each serum miRNA signal was normalized to the ratio of the adjusted Int-con.

For the miRNA analysis of cell lysate samples, 250 ng of total RNA from biopsied samples was used. In addition, 300 µL of cell-cultured supernatant was subjected to microarray analysis.

**t*-SNE***

The perplexity parameter in *t*-SNE analysis [2, 3] indicates the effective number of neighbors, resulting in a *t*-SNE plot that may differ depending on the setting. In addition, iterations are important for obtaining a stable result. Grouping by *t*-SNE analysis was the same regardless of the value of perplexity parameters (10, 25, and 50) and max iterations (100, 500, and 5000). In this study, the perplexities of 25 and 5000 iterations were optimized. All missing values in the data were replaced with the minimum value of the respective miRNA in the analysis set minus 0.1.

***WGCNA***

WGCNA defines a weighted adjacency matrix and clusters genes using 1–topological overlap measure (TOM) as a dissimilarity measure [4]. Gene modules were defined as branches of the resulting cluster tree using a dynamic branch-cutting algorithm. Eigengenes for each clustering module were used as representative expression patterns of the miRNA signals in each module. Module–trait associations were investigated between these eigengenes and tumor factors, including the presence of PBca, CA19-9 level, UICC clinical TNM factors, and UICC clinical stages as PBca features and time to serum preparation and circulating platelet count as disturbance factors in c-miRNA measurement. To determine the soft thresholding power, the scale-free fit index and mean connectivity were examined (Supplementary Figure S1), and soft thresholds of 4, 5, and 6 were selected as candidates. A microRNA dendrogram and modules were obtained using the TOM by changing the minimum cluster size (10, 20, 30) and deep slit (0, 1, 2, 3, 4). After dynamic merging of the modules, the best clustering was selected.

***Clustering and heatmap***

We conducted clustering analysis using the nearest-neighbors method with Euclidean distance in a two-dimensional space based on miRNA expression data. The results are shown in a heatmap and clustering dendrogram for the miRNA expression data and participants.

***R packages***

Statistical analyses were performed using R version 4.0.3 (R Foundation for Statistical Computing, http://www.R-project.org) and the compute.es 0.2-5 / tidyverse 1.3.1 / Rtsne 0.15/ WGCNA 1.70.3 / MASS 7.3.53 / pROC 1.18.0. / beeswarm 0.4.0 / heatmap.3 (Griffith, 2014) biostar tutorial, <https://github.com/obigriffith/biostar-tutorials/blob/master/Heatmaps/heatmap.3.R>).

***microRNA expression analysis of cultured cell lines***

KP-2, Capan-1, Suit-2, Miapaca-2, and CFPAC-1 cells were grown in 10 cm dishes in duplicate for 24 h, whereas Panc-1, SW1990, and KP-4 cells were grown for 48 h. Subsequently, the culture medium was replaced with serum-free medium, and the cells were incubated for an additional 24 h. A total of 300 μL of the culture medium and the entire cell content were collected for RNA extraction. RNA was extracted from cell lysate samples using the miRVana™ Paris™ RNA and Native Protein Purification Kit (Thermo Fisher Scientific, Waltham, MA, USA), whereas RNA was extracted from supernatant samples using the 3D-Gene^®^ RNA extraction reagent (Toray Industries, Inc.). Next, 250 ng of RNA extracted from each cell lysate and half of the RNA extracted from each supernatant were used for microarray analysis using the method described above. Microarray data were obtained as described above, followed by global normalization of the obtained miRNA data.

**Supplemental References**

1. Shimomura A, Shiino S, Kawauchi J, Takizawa S, Sakamoto H, Matsuzaki J, et al. Novel combination of serum microRNA for detecting breast cancer in the early stage. Cancer Sci. 2016;107:326-34.

2. van der Maaten L, Hinton G. Visualizing data using t-SNE. J Mach Learn Res. 2008;9:85.

3. van der Maaten L. Accelerating t-SNE using tree-based algorithms. J Mach Learn Res. 2014;15:3221-45.

4. Langfelder P, Horvath S. WGCNA: an R package for weighted correlation network analysis. BMC Bioinformatics. 2008;9:559.

**Supplementary Figures**

**Supplementary Figure S1. Performance of discriminants with three to six miRNA variables.** Performance of miRNA discriminants with three to six miRNA variables in the exploratory set. The x-axis is the mean sensitivity of 50× cross-validation; the y-axis is the mean specificity of 50× cross-validation. Numbers on the top of each plot represent the number of combined miRNA variables.


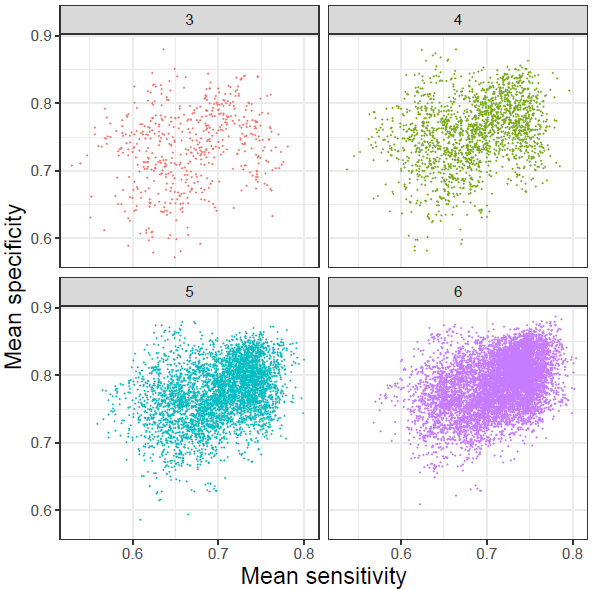

**Supplementary Figure S2.** ***t*-SNE plot of comprehensive miRNAs in healthy control serum.** *t*-SNE plot based on 397 miRNAs without NA values from 210 heathy controls included in Groups 1, 4, and 5. Group categorization is shown in Figure 2c.

a.


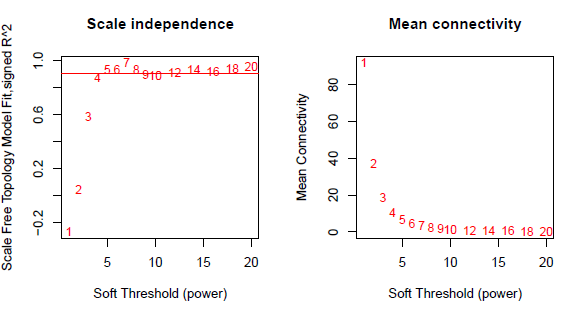


b.


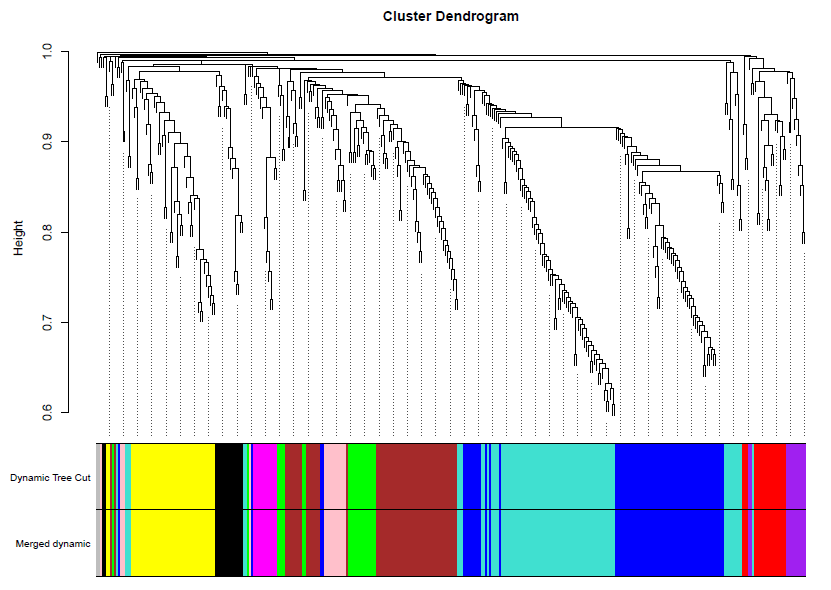


**Supplementary Figure S3.** **WGCNA optimization of 357 robust miRNAs.** (a) Scale independency and mean connectivity dependent on soft threshold. x-axis: soft-threshold power. y-axis: scale-free topology model fit (left) or mean connectivity (right). (b) Cluster dendrogram of the coexpression network modules on the condition of soft power 5, minimum module 10, and deep slit 3.


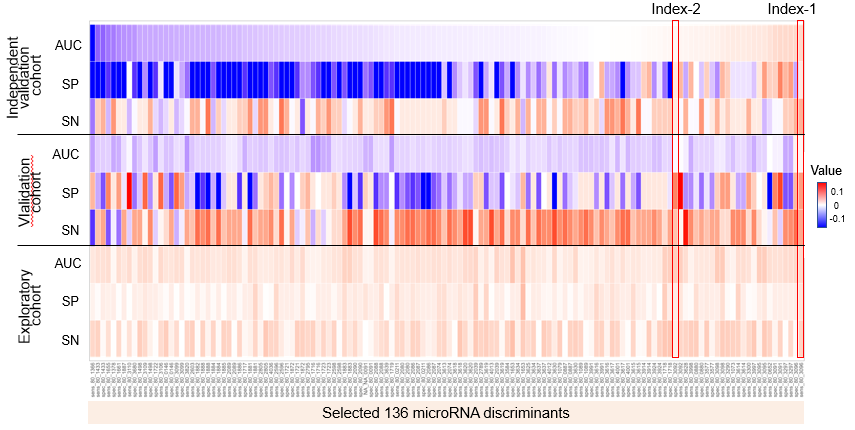


**Supplementary Figure S4. Performance of 136 miRNA discriminants.** The performance of miRNA discriminants in the three evaluation sets is shown in different colors. Sensitivity or specificity is shown as the value subtracted by 0.8, with the color gradient ranging from blue (–0.1) to red (+0.1). The AUC was calculated as the value subtracted from that of CA19-9 in each set. The color gradient was the same as that for sensitivity/specificity. miRNA discriminants with values greater than 0 for sensitivity/specificity/AUC in all sets were selected as effective candidates and are shown in Index-1 or Index-2.

**Supplementary Table S1.** **Time until sample freezing.**

| Group | Class | Average time (h:mm) | | | |
| --- | --- | --- | --- | --- | --- |
|  |  | Average | SD | Minimum | Max |
| 3 | HC | - | - | - | - |
|  | PBca | 0:51 | 0:06 | 0:44 | 1:13 |
| 4 | HC | 1:02 | 0:08 | 0:46 | 1:25 |
|  | PBca | 0:53 | 0:06 | 0:45 | 1:15 |
| 5 | HC | 1:03 | 0:06 | 0:53 | 1:22 |
|  | PBca | 0:57 | 0:12 | 0:45 | 1:39 |

Abbreviations: HC, healthy control; PBca, pancreatobiliary cancer; SD, standard deviation

**Supplementary Table S2.** **Clinical background.**

| Variable | | | Exploratory set | | | | | | | Validation set | | | | | | | Independent validation set | | | |
| --- | --- | --- | --- | --- | --- | --- | --- | --- | --- | --- | --- | --- | --- | --- | --- | --- | --- | --- | --- | --- |
|  |  |  | PBca  (n = 134) | | | Healthy participants  (n = 150) | | | PBca  (n = 47) | | | | Healthy participants  (n = 50) | | PBca  (n = 46) | | | | | Healthy participants  (n = 50) |
|  |  |  | n (%) | | | | | | | | | | | | | | | | | |
| Age, years | Median [IQR] | 70  [61–76] | | 70  [66–74] | | | | 67  [60–72] | | | 71  [65–75] | | | | | 73  [66–80] | | 69  [63–72] | | |
| Sex | Men | 78 (58.2) | | | 95 (63.3) | | 24 (51.1) | | | | | 32 (64.0) | | 24 (52.2) | | | | | 34 (68.0) | |
| Platelets, ×10^4^/mm^3^ | Median [IQR] | 25.45 [17.25–29.93] (n = 16) | | |  | | 22.2  [18.50–26.35] (n = 31) | | | | |  | | 22.30 [18.50–29.45] (n = 46) | | | | | 24.15 [19.65–27.80] (n = 50) | |
| CA19-9, U/mL | Median [IQR] | 153.4  [22.7–1170] | | | 9  [5.6–15.0] | | 837 [106–5180] | | | | | 8.2  [5.1–17.2] | | 680 [61.6–5960] | | | | | 6.6  [3.1–13.6] | |
| UICC stage | 0, I, II | 52 (38.8) | | |  | | 8 (17.0) | | | | |  | | 8 (17.4) | | | | |  | |
|  | III, IV | 82 (61.2) | | |  | | 39 (83.0) | | | | |  | | 38 (82.6) | | | | |  | |
| Pancreatic cancer |  | 98 (73.1) | | |  | | 35 (74.5) | | | | |  | | 36 (78.3) | | | | |  | |
| Primary site | Head | 52 (38.8) | | |  | | 13 (27.7) | | | | |  | | 23 (50.0) | | | | |  | |
|  | Body | 26 (19.4) | | |  | | 8 (17.0) | | | | |  | | 10 (21.7) | | | | |  | |
|  | Tail | 20 (14.9) | | |  | | 14 (29.8) | | | | |  | | 3 (6.5) | | | | |  | |
| Biliary tract cancer |  | 36 (26.9) | | |  | | 12 (25.5) | | | | |  | | 10 (21.7) | | | | |  | |
| Primary site | Intrahepatic | 14 (10.5) | | |  | | 4 (8.5) | | | | |  | | 4 (8.7) | | | | |  | |
|  | Gall bladder | 6 (4.5) | | |  | | 3 (6.4) | | | | |  | | 5 (10.9) | | | | |  | |
|  | Hilar | 5 (3.7) | | |  | | 2 (4.3) | | | | |  | | 1 (2.2) | | | | |  | |
|  | Distal | 6 (4.5) | | |  | | 2 (4.3) | | | | |  | | 0 | | | | |  | |
|  | Vater | 5 (3.7) | | |  | | 1 (2.0) | | | | |  | | 0 | | | | |  | |
| Institution for PBca | ACC | 22 | | |  | | - | | | | |  | | - | | | | |  | |
|  | ASA | 4 | | |  | | - | | | | |  | | 1 | | | | |  | |
|  | HCC | 30 | | |  | | - | | | | |  | | 1 | | | | |  | |
|  | KCC | 50 | | |  | | 16 | | | | |  | | 8 | | | | |  | |
|  | KYU | 12 | | |  | | 31 | | | | |  | | - | | | | |  | |
|  | NCC | 16 | | |  | | - | | | | |  | | 27 | | | | |  | |
|  | ONO | - | | |  | | - | | | | |  | | 5 | | | | |  | |
|  | SYO | - | | |  | | - | | | | |  | | 4 | | | | |  | |
| Institution for HC | HKT | 20 | | |  | | - | | | | |  | | - | | | | |  | |
|  | SKN | 33 | | |  | | 17 | | | | |  | | - | | | | |  | |
|  | SMI | 50 | | |  | | - | | | | |  | | - | | | | |  | |
|  | TRY | 13 | | |  | | - | | | | |  | | - | | | | |  | |
|  | YMC | 34 | | |  | | 33 | | | | |  | | 50 | | | | |  | |

Abbreviations: HC, healthy control; PBca, pancreatobiliary cancer; IQR, interquartile range; CA19-9, carbohydrate antigen 19-9; UICC, Union for International Cancer Control.

ACC, Aichi Cancer Center; ASA, Asahikawa Medical University Hospital; HCC, Hyogo Cancer Center; KCC, Kanagawa Cancer Center; KYU, Kyusyu University Hospital; NCC, National Cancer Center East Hospital; ONO, JA Onomichi General Hospital; SHO, Showa University Hospital; HKT, Hakata Clinic; SKN, Sekino Rinsyo-Yakuri Clinic; SMI, Sumida Hospital; TRY, Toray Industries, Inc.; YMC, Yokohama Minoru Clinic.

**Supplementary Table S3. Features of microRNA probes.**

| Probe | Exploratory set  (n = 284) | Healthy participants of exploratory set  (n = 150) | | Healthy participants of time-course effects study  (n = 150) | | Slope difference | Probe type | WGCNA module | Ridge regression coefficient |
| --- | --- | --- | --- | --- | --- | --- | --- | --- | --- |
|  | Effective call rate | r^2^ | Slope | r^2^ | Slope |  |  |  |  |
| hsa-miR-1268a | 100 | 0.95 | –1 | 0.91 | –1.01 | 0.01 | Robust | Turquoise | –0.21 |
| hsa-miR-1343-5p | 100 | 0.97 | –1.08 | 0.94 | –1.08 | 0 | Robust | Blue | 0.3 |
| hsa-miR-1469 | 100 | 0.92 | –0.86 | 0.87 | –0.93 | 0.07 | Robust | Yellow | –0.14 |
| hsa-miR-1908-5p | 100 | 0.96 | –0.98 | 0.93 | –1.04 | 0.06 | Robust | Turquoise | –0.14 |
| hsa-miR-3940-5p | 100 | 0.96 | –0.91 | 0.93 | –0.89 | 0.02 | Robust | Yellow | –0.22 |
| hsa-miR-4466 | 100 | 0.96 | –0.92 | 0.94 | –0.92 | 0 | Robust | Yellow | –0.21 |
| hsa-miR-4632-5p | 100 | 0.95 | –1.03 | 0.93 | –1.05 | 0.02 | Robust | Brown | 0.18 |
| hsa-miR-4665-5p | 100 | 0.92 | –0.89 | 0.89 | –0.88 | 0.01 | Robust | Turquoise | 0.23 |
| hsa-miR-4687-3p | 100 | 0.97 | –1.15 | 0.95 | –1.15 | 0 | Robust | Turquoise | –0.14 |
| hsa-miR-4734 | 100 | 0.94 | –0.77 | 0.91 | –0.78 | 0.01 | Robust | Yellow | –0.16 |
| hsa-miR-665 | 100 | 0.86 | –1.06 | 0.7 | –1.01 | 0.05 | Robust | Magenta | –0.14 |
| hsa-miR-6787-5p | 100 | 0.94 | –1.1 | 0.89 | –1.1 | 0 | Robust | Brown | 0.2 |
| hsa-miR-6803-5p | 100 | 0.97 | –0.97 | 0.96 | –0.95 | 0.02 | Robust | Yellow | –0.28 |
| hsa-miR-8069 | 100 | 0.96 | –0.9 | 0.9 | –0.85 | 0.05 | Robust | Yellow | –0.17 |
| hsa-miR-8085 | 100 | 0.94 | –1.16 | 0.88 | –1.12 | 0.04 | Robust | Magenta | –0.15 |
| hsa-miR-92b-5p | 100 | 0.96 | –1.15 | 0.95 | –1.17 | 0.02 | Robust | Blue | 0.15 |
| hsa-miR-149-3p | 100 | 0.97 | –0.99 | 0.93 | –1.03 | 0.04 | Internal control | Brown | 0.06 |
| hsa-miR-2861 | 100 | 0.97 | –1.02 | 0.96 | –0.97 | 0.05 | Internal control | Brown | –0.15 |
| hsa-miR-4463 | 100 | 0.98 | –0.98 | 0.97 | –1 | 0.02 | Internal control | Green |  |
| hsa-miR-10226 | 99.7 | 0.66 | –1.21 | 0.62 | –1.14 | 0.07 | Robust | Blue | 0 |
| hsa-miR-10392-5p | 100 | 0.81 | –1.06 | 0.69 | –1.07 | 0.01 | Robust | Turquoise | –0.02 |
| hsa-miR-10394-3p | 100 | 0.86 | –0.86 | 0.82 | –0.86 | 0 | Robust | Green |  |
| hsa-miR-10396a-5p | 100 | 0.98 | –0.89 | 0.95 | –0.92 | 0.03 | Robust | Yellow | –0.1 |
| hsa-miR-10396b-3p | 99.7 | 0.83 | –0.88 | 0.82 | –0.97 | 0.09 | Robust | Gray |  |
| hsa-miR-10396b-5p | 100 | 0.95 | –0.84 | 0.89 | –0.85 | 0.01 | Robust | Turquoise | –0.03 |
| hsa-miR-10398-3p | 100 | 0.84 | –0.94 | 0.84 | –0.95 | 0.01 | Robust | Pink |  |
| hsa-miR-10400-3p | 100 | 0.88 | –1.16 | 0.88 | –1.07 | 0.09 | Robust | Blue | –0.03 |
| hsa-miR-10401-3p | 100 | 0.69 | –0.94 | 0.72 | –0.96 | 0.02 | Robust | Blue | 0.03 |
| hsa-miR-10401-5p | 100 | 0.95 | –1.07 | 0.94 | –1.07 | 0 | Robust | Pink |  |
| hsa-miR-10526-3p | 100 | 0.9 | –1.17 | 0.76 | –1.2 | 0.03 | Robust | Turquoise | –0.06 |
| hsa-miR-10527-5p | 100 | 0.71 | –0.94 | 0.73 | –0.95 | 0.01 | Robust | Blue | 0.03 |
| hsa-miR-11181-3p | 100 | 0.92 | –1.19 | 0.76 | –1.19 | 0 | Robust | Magenta | –0.09 |
| hsa-miR-1181 | 100 | 0.83 | –0.72 | 0.65 | –0.7 | 0.02 | Robust | Blue | 0.01 |
| hsa-miR-1182 | 98 | 0.81 | –1.08 | 0.65 | –1.05 | 0.03 | Unrobust |  |  |
| hsa-miR-1185-1-3p | 100 | 0.84 | –1.12 | 0.82 | –1.15 | 0.03 | Robust | Brown | –0.05 |
| hsa-miR-1193 | 100 | 0.85 | –0.92 | 0.77 | –0.95 | 0.03 | Robust | Brown | –0.1 |
| hsa-miR-1199-5p | 100 | 0.72 | –0.79 | 0.68 | –0.7 | 0.09 | Robust | Yellow | –0.01 |
| hsa-miR-1202 | 100 | 0.86 | –1.19 | 0.83 | –1.22 | 0.03 | Robust | Brown | 0 |
| hsa-miR-1203 | 100 | 0.84 | –0.99 | 0.73 | –0.98 | 0.01 | Robust | Black |  |
| hsa-miR-1207-5p | 100 | 0.94 | –1.13 | 0.92 | –1.13 | 0 | Robust | Blue | 0 |
| hsa-miR-12114 | 100 | 0.9 | –1.2 | 0.81 | –1.16 | 0.04 | Robust | Brown | –0.03 |
| hsa-miR-12115 | 99.7 | 0.63 | –1.29 | 0.58 | –1.2 | 0.09 | Robust | Blue | 0.01 |
| hsa-miR-12118 | 100 | 0.95 | –1.08 | 0.95 | –0.99 | 0.09 | Robust | Blue | 0.07 |
| hsa-miR-12119 | 98 | 0.76 | –1.02 | 0.82 | –1.1 | 0.08 | Unrobust |  |  |
| hsa-miR-12120 | 100 | 0.93 | –1.28 | 0.85 | –1.28 | 0 | Robust | Turquoise | 0.01 |
| hsa-miR-1225-5p | 100 | 0.95 | –1.1 | 0.92 | –1.11 | 0.01 | Robust | Brown | 0.07 |
| hsa-miR-1227-5p | 100 | 0.96 | –1 | 0.89 | –1.03 | 0.03 | Robust | Turquoise | 0.03 |
| hsa-miR-1228-3p | 99.3 | 0.69 | –0.68 | 0.56 | –0.71 | 0.03 | Robust | Red |  |
| hsa-miR-1228-5p | 100 | 0.97 | –1.06 | 0.97 | –1.02 | 0.04 | Robust | Yellow | –0.01 |
| hsa-miR-1231 | 100 | 0.84 | –0.97 | 0.76 | –0.94 | 0.03 | Robust | Turquoise | –0.03 |
| hsa-miR-1233-5p | 100 | 0.89 | –1.03 | 0.84 | –1.06 | 0.03 | Robust | Yellow | 0 |
| hsa-miR-1236-5p | 100 | 0.89 | –1.21 | 0.76 | –1.28 | 0.07 | Robust | Brown | 0.02 |
| hsa-miR-1237-5p | 100 | 0.96 | –0.9 | 0.93 | –0.94 | 0.04 | Robust | Yellow | –0.09 |
| hsa-miR-1247-3p | 100 | 0.88 | –0.89 | 0.74 | –0.85 | 0.04 | Robust | Brown | 0.04 |
| hsa-miR-1249-5p | 100 | 0.68 | –1.01 | 0.73 | –1.07 | 0.06 | Robust | Blue | 0.01 |
| hsa-miR-1268b | 100 | 0.93 | –0.97 | 0.92 | –0.95 | 0.02 | Robust | Turquoise | –0.13 |
| hsa-miR-1273h-5p | 98 | 0.7 | –0.94 | 0.56 | –1.02 | 0.08 | Unrobust |  |  |
| hsa-miR-128-1-5p | 100 | 0.77 | –0.96 | 0.66 | –0.89 | 0.07 | Robust | Black |  |
| hsa-miR-1292-3p | 100 | 0.76 | –0.69 | 0.69 | –0.7 | 0.01 | Robust | Blue | 0.07 |
| hsa-miR-1303 | 99.3 | 0.88 | –1.18 | 0.74 | –1.17 | 0.01 | Robust | Purple |  |
| hsa-miR-1307-3p | 100 | 0.81 | –1.08 | 0.62 | –1.11 | 0.03 | Robust | Turquoise | 0.03 |
| hsa-miR-1343-3p | 100 | 0.83 | –0.83 | 0.73 | –0.85 | 0.02 | Robust | Brown | 0 |
| hsa-miR-139-3p | 99 | 0.56 | –0.8 | 0.6 | –0.89 | 0.09 | Robust | Blue | 0.01 |
| hsa-miR-1587 | 100 | 0.74 | –1.36 | 0.61 | –1.27 | 0.09 | Robust | Blue | –0.01 |
| hsa-miR-1909-3p | 100 | 0.91 | –0.95 | 0.89 | –0.92 | 0.03 | Robust | Black |  |
| hsa-miR-1910-5p | 98.7 | 0.76 | –0.77 | 0.73 | –0.8 | 0.03 | Unrobust |  |  |
| hsa-miR-1914-3p | 100 | 0.9 | –1.09 | 0.85 | –1.07 | 0.02 | Robust | Brown | 0.08 |
| hsa-miR-1915-3p | 100 | 0.94 | –0.94 | 0.87 | –0.93 | 0.01 | Robust | Yellow | –0.13 |
| hsa-miR-1915-5p | 99.3 | 0.52 | –1.01 | 0.52 | –0.97 | 0.04 | Robust | Yellow | 0.01 |
| hsa-miR-197-5p | 100 | 0.87 | –1.11 | 0.59 | –1.21 | 0.1 | Robust | Blue | –0.03 |
| hsa-miR-198 | 98.3 | 0.84 | –1.19 | 0.73 | –1.15 | 0.04 | Unrobust |  |  |
| hsa-miR-2110 | 99.3 | 0.84 | –0.89 | 0.67 | –0.9 | 0.01 | Robust | Green |  |
| hsa-miR-211-3p | 100 | 0.72 | –0.82 | 0.65 | –0.82 | 0 | Robust | Brown | 0.04 |
| hsa-miR-2278 | 99.3 | 0.69 | –0.77 | 0.66 | –0.85 | 0.08 | Robust | Pink |  |
| hsa-miR-2467-3p | 99.3 | 0.79 | –1.07 | 0.62 | –1.05 | 0.02 | Robust | Magenta | –0.03 |
| hsa-miR-296-3p | 98 | 0.74 | –0.79 | 0.54 | –0.79 | 0 | Unrobust |  |  |
| hsa-miR-3085-5p | 99 | 0.81 | –1.18 | 0.72 | –1.16 | 0.02 | Robust | Red |  |
| hsa-miR-30c-1-3p | 100 | 0.89 | –1.24 | 0.82 | –1.31 | 0.07 | Robust | Brown | 0.04 |
| hsa-miR-3126-5p | 99.3 | 0.8 | –0.87 | 0.53 | –0.89 | 0.02 | Robust | Purple |  |
| hsa-miR-3135b | 100 | 0.74 | –1.05 | 0.7 | –1.01 | 0.04 | Robust | Blue | 0 |
| hsa-miR-3147 | 100 | 0.92 | –1.15 | 0.87 | –1.11 | 0.04 | Robust | Magenta | –0.11 |
| hsa-miR-3150a-3p | 98.3 | 0.81 | –1.22 | 0.64 | –1.15 | 0.07 | Unrobust |  |  |
| hsa-miR-3151-5p | 99.7 | 0.79 | –1.16 | 0.72 | –1.12 | 0.04 | Robust | Blue | 0.03 |
| hsa-miR-3154 | 100 | 0.81 | –0.98 | 0.85 | –1.06 | 0.08 | Robust | Brown | 0.02 |
| hsa-miR-3162-5p | 100 | 0.81 | –1.28 | 0.79 | –1.36 | 0.08 | Robust | Brown | 0.03 |
| hsa-miR-3177-3p | 98 | 0.74 | –0.86 | 0.56 | –0.96 | 0.1 | Unrobust |  |  |
| hsa-miR-3178 | 100 | 0.91 | –0.89 | 0.83 | –0.85 | 0.04 | Robust | Yellow | –0.07 |
| hsa-miR-3180 | 100 | 0.96 | –0.97 | 0.96 | –0.99 | 0.02 | Robust | Green |  |
| hsa-miR-3180-3p | 100 | 0.95 | –0.96 | 0.85 | –0.94 | 0.02 | Robust | Turquoise | 0.07 |
| hsa-miR-3184-5p | 100 | 0.75 | –0.94 | 0.56 | –0.87 | 0.07 | Robust | Black |  |
| hsa-miR-3185 | 100 | 0.88 | –1.04 | 0.75 | –1 | 0.04 | Robust | Turquoise | 0.03 |
| hsa-miR-3187-3p | 100 | 0.91 | –0.94 | 0.67 | –0.98 | 0.04 | Robust | Turquoise | –0.01 |
| hsa-miR-3187-5p | 99.7 | 0.86 | –1.01 | 0.67 | –1.03 | 0.02 | Robust | Turquoise | 0.01 |
| hsa-miR-3188 | 100 | 0.86 | –0.97 | 0.85 | –1.01 | 0.04 | Robust | Pink |  |
| hsa-miR-3190-3p | 99 | 0.68 | –1.31 | 0.69 | –1.31 | 0 | Robust | Blue | 0.05 |
| hsa-miR-3191-3p | 99.7 | 0.92 | –1.03 | 0.75 | –1 | 0.03 | Robust | Purple |  |
| hsa-miR-3195 | 100 | 0.91 | –0.94 | 0.88 | –0.99 | 0.05 | Robust | Green |  |
| hsa-miR-3196 | 100 | 0.97 | –0.9 | 0.93 | –0.91 | 0.01 | Robust | Yellow | 0 |
| hsa-miR-3197 | 100 | 0.9 | –0.91 | 0.82 | –0.93 | 0.02 | Robust | Turquoise | –0.05 |
| hsa-miR-320a-3p | 99.3 | 0.81 | –1.05 | 0.77 | –1.14 | 0.09 | Robust | Purple |  |
| hsa-miR-328-5p | 100 | 0.98 | –1.02 | 0.95 | –1.01 | 0.01 | Robust | Yellow | –0.15 |
| hsa-miR-3620-5p | 100 | 0.9 | –1.1 | 0.79 | –1.07 | 0.03 | Robust | Blue | –0.02 |
| hsa-miR-3621 | 100 | 0.83 | –0.84 | 0.91 | –0.87 | 0.03 | Robust | Blue | 0.03 |
| hsa-miR-3622a-5p | 100 | 0.91 | –1.18 | 0.84 | –1.11 | 0.07 | Robust | Blue | 0.1 |
| hsa-miR-3652 | 100 | 0.89 | –1.04 | 0.82 | –1.05 | 0.01 | Robust | Brown | 0.04 |
| hsa-miR-3663-5p | 99.7 | 0.65 | –1.16 | 0.61 | –1.17 | 0.01 | Robust | Blue | 0 |
| hsa-miR-3665 | 100 | 0.95 | –0.87 | 0.89 | –0.82 | 0.05 | Robust | Yellow | –0.13 |
| hsa-miR-371a-5p | 100 | 0.85 | –1.06 | 0.72 | –1.06 | 0 | Robust | Brown | –0.07 |
| hsa-miR-371b-5p | 100 | 0.8 | –1.04 | 0.53 | –1.09 | 0.05 | Robust | Turquoise | 0.05 |
| hsa-miR-3911 | 98.3 | 0.86 | –1.19 | 0.72 | –1.16 | 0.03 | Unrobust |  |  |
| hsa-miR-3917 | 99.7 | 0.82 | –1.06 | 0.67 | –1.05 | 0.01 | Robust | Blue | 0.03 |
| hsa-miR-3928-3p | 100 | 0.85 | –1 | 0.7 | –1.03 | 0.03 | Robust | Brown | 0.12 |
| hsa-miR-3934-5p | 94 | 0.7 | –1.03 | 0.61 | –1.09 | 0.06 | Unrobust |  |  |
| hsa-miR-3937 | 100 | 0.9 | –1.09 | 0.8 | –1.06 | 0.03 | Robust | Turquoise | –0.06 |
| hsa-miR-4253 | 97.3 | 0.82 | –0.86 | 0.66 | –0.85 | 0.01 | Unrobust |  |  |
| hsa-miR-4258 | 100 | 0.65 | –0.59 | 0.57 | –0.58 | 0.01 | Robust | Black |  |
| hsa-miR-4259 | 94.7 | 0.81 | –0.87 | 0.63 | –0.92 | 0.05 | Unrobust |  |  |
| hsa-miR-4260 | 97.7 | 0.75 | –0.94 | 0.64 | –0.96 | 0.02 | Unrobust |  |  |
| hsa-miR-4265 | 99.7 | 0.61 | –1.12 | 0.51 | –1.14 | 0.02 | Robust | Blue | 0.01 |
| hsa-miR-4270 | 100 | 0.95 | –0.9 | 0.91 | –0.9 | 0 | Robust | Pink |  |
| hsa-miR-4271 | 100 | 0.92 | –1.12 | 0.88 | –1.13 | 0.01 | Robust | Blue | 0 |
| hsa-miR-4276 | 100 | 0.82 | –0.91 | 0.66 | –0.87 | 0.04 | Robust | Yellow | –0.03 |
| hsa-miR-4281 | 100 | 0.95 | –1.01 | 0.88 | –0.98 | 0.03 | Robust | Brown | –0.03 |
| hsa-miR-4298 | 100 | 0.91 | –1.1 | 0.8 | –1.1 | 0 | Robust | Magenta | –0.1 |
| hsa-miR-4322 | 100 | 0.78 | –1.11 | 0.59 | –1.17 | 0.06 | Robust | Turquoise | 0.05 |
| hsa-miR-4327 | 100 | 0.89 | –1.08 | 0.77 | –1.12 | 0.04 | Robust | Turquoise | 0.02 |
| hsa-miR-4428 | 100 | 0.86 | –1.29 | 0.7 | –1.26 | 0.03 | Robust | Turquoise | –0.04 |
| hsa-miR-4430 | 100 | 0.85 | –1.02 | 0.82 | –1.06 | 0.04 | Robust | Brown | 0.03 |
| hsa-miR-4433a-3p | 100 | 0.94 | –1.11 | 0.93 | –1.11 | 0 | Robust | Green |  |
| hsa-miR-4433b-3p | 100 | 0.94 | –1.12 | 0.88 | –1.1 | 0.02 | Robust | Green |  |
| hsa-miR-4442 | 100 | 0.77 | –0.82 | 0.7 | –0.84 | 0.02 | Robust | Brown | 0.01 |
| hsa-miR-4443 | 100 | 0.79 | –1.13 | 0.77 | –1.14 | 0.01 | Robust | Brown | –0.02 |
| hsa-miR-4446-3p | 100 | 0.86 | –0.85 | 0.85 | –0.78 | 0.07 | Robust | Yellow | –0.03 |
| hsa-miR-4447 | 100 | 0.92 | –1.19 | 0.89 | –1.16 | 0.03 | Robust | Green |  |
| hsa-miR-4449 | 100 | 0.71 | –0.83 | 0.74 | –0.87 | 0.04 | Robust | Blue | –0.01 |
| hsa-miR-4462 | 99.7 | 0.84 | –1.31 | 0.85 | –1.21 | 0.1 | Robust | Turquoise | 0.01 |
| hsa-miR-4467 | 100 | 0.87 | –1.02 | 0.73 | –1.05 | 0.03 | Robust | Turquoise | 0.01 |
| hsa-miR-4472 | 100 | 0.88 | –1.16 | 0.84 | –1.21 | 0.05 | Robust | Green |  |
| hsa-miR-4478 | 100 | 0.86 | –1.07 | 0.72 | –1.05 | 0.02 | Robust | Magenta | –0.11 |
| hsa-miR-4479 | 98.7 | 0.87 | –1.03 | 0.76 | –1.02 | 0.01 | Unrobust |  |  |
| hsa-miR-4483 | 98.7 | 0.85 | –1.23 | 0.89 | –1.28 | 0.05 | Unrobust |  |  |
| hsa-miR-4486 | 100 | 0.91 | –1.17 | 0.81 | –1.09 | 0.08 | Robust | Turquoise | –0.02 |
| hsa-miR-4487 | 99.3 | 0.79 | –0.97 | 0.74 | –1.06 | 0.09 | Robust | Red |  |
| hsa-miR-4488 | 100 | 0.94 | –0.91 | 0.94 | –0.89 | 0.02 | Robust | Yellow | –0.07 |
| hsa-miR-4492 | 100 | 0.91 | –0.95 | 0.86 | –0.94 | 0.01 | Robust | Turquoise | 0.03 |
| hsa-miR-4498 | 99.7 | 0.66 | –1.22 | 0.51 | –1.25 | 0.03 | Robust | Blue | 0.02 |
| hsa-miR-4505 | 100 | 0.88 | –1.1 | 0.76 | –1.13 | 0.03 | Robust | Turquoise | 0.03 |
| hsa-miR-4507 | 100 | 0.76 | –1.29 | 0.63 | –1.23 | 0.06 | Robust | Blue | –0.01 |
| hsa-miR-4508 | 100 | 0.95 | –0.91 | 0.93 | –0.92 | 0.01 | Robust | Yellow | –0.02 |
| hsa-miR-4513 | 100 | 0.8 | –0.87 | 0.74 | –0.95 | 0.08 | Robust | Brown | –0.03 |
| hsa-miR-4516 | 100 | 0.91 | –0.87 | 0.93 | –0.89 | 0.02 | Robust | Green |  |
| hsa-miR-4525 | 100 | 0.89 | –1.09 | 0.84 | –1.12 | 0.03 | Robust | Pink |  |
| hsa-miR-4526 | 96 | 0.82 | –1 | 0.7 | –1.05 | 0.05 | Unrobust |  |  |
| hsa-miR-4530 | 100 | 0.92 | –1.1 | 0.82 | –1.07 | 0.03 | Robust | Pink |  |
| hsa-miR-4533 | 99.3 | 0.85 | –0.92 | 0.57 | –0.92 | 0 | Robust | Red |  |
| hsa-miR-4534 | 100 | 0.57 | –1.02 | 0.63 | –1.06 | 0.04 | Robust | Blue | 0 |
| hsa-miR-4535 | 100 | 0.76 | –1.11 | 0.77 | –1.01 | 0.1 | Robust | Blue | 0.02 |
| hsa-miR-4539 | 100 | 0.71 | –1.12 | 0.52 | –1.13 | 0.01 | Robust | Turquoise | 0.03 |
| hsa-miR-4634 | 100 | 0.93 | –0.91 | 0.81 | –0.87 | 0.04 | Robust | Yellow | –0.06 |
| hsa-miR-4638-3p | 99.3 | 0.61 | –0.91 | 0.51 | –0.92 | 0.01 | Robust | Blue | –0.01 |
| hsa-miR-4638-5p | 100 | 0.67 | –1.22 | 0.81 | –1.24 | 0.02 | Robust | Blue | 0.03 |
| hsa-miR-4640-5p | 100 | 0.98 | –1.03 | 0.94 | –1.03 | 0 | Robust | Pink |  |
| hsa-miR-4648 | 99.7 | 0.7 | –1.34 | 0.59 | –1.27 | 0.07 | Robust | Blue | –0.03 |
| hsa-miR-4651 | 100 | 0.97 | –0.95 | 0.93 | –0.96 | 0.01 | Robust | Blue | 0.1 |
| hsa-miR-4656 | 100 | 0.84 | –1.02 | 0.65 | –1.07 | 0.05 | Robust | Magenta | –0.09 |
| hsa-miR-4665-3p | 100 | 0.84 | –0.76 | 0.81 | –0.79 | 0.03 | Robust | Turquoise | 0.04 |
| hsa-miR-4667-5p | 100 | 0.8 | –1.03 | 0.81 | –0.96 | 0.07 | Robust | Brown | 0 |
| hsa-miR-4669 | 100 | 0.79 | –0.99 | 0.68 | –1.03 | 0.04 | Robust | Brown | 0.05 |
| hsa-miR-4674 | 100 | 0.85 | –0.76 | 0.7 | –0.75 | 0.01 | Robust | Yellow | –0.05 |
| hsa-miR-4675 | 100 | 0.82 | –0.87 | 0.77 | –0.87 | 0 | Robust | Green |  |
| hsa-miR-4689 | 100 | 0.88 | –1 | 0.9 | –1.02 | 0.02 | Robust | Brown | –0.04 |
| hsa-miR-4690-5p | 100 | 0.78 | –1.1 | 0.61 | –1.15 | 0.05 | Robust | Turquoise | 0.02 |
| hsa-miR-4695-5p | 100 | 0.89 | –1.07 | 0.76 | –1.06 | 0.01 | Robust | Turquoise | –0.02 |
| hsa-miR-4697-5p | 100 | 0.94 | –1.04 | 0.93 | –1.03 | 0.01 | Robust | Green |  |
| hsa-miR-4698 | 98.7 | 0.78 | –1.03 | 0.5 | –0.97 | 0.06 | Unrobust |  |  |
| hsa-miR-4706 | 100 | 0.91 | –0.98 | 0.85 | –1.03 | 0.05 | Robust | Brown | 0 |
| hsa-miR-4707-3p | 99.3 | 0.79 | –1 | 0.73 | –1.02 | 0.02 | Robust | Black |  |
| hsa-miR-4707-5p | 100 | 0.91 | –0.96 | 0.77 | –0.96 | 0 | Robust | Turquoise | 0.1 |
| hsa-miR-4710 | 99.7 | 0.82 | –1.12 | 0.73 | –1.09 | 0.03 | Robust | Red |  |
| hsa-miR-4717-3p | 98 | 0.78 | –1 | 0.58 | –1.02 | 0.02 | Unrobust |  |  |
| hsa-miR-4721 | 100 | 0.75 | –0.92 | 0.51 | –0.83 | 0.09 | Robust | Brown | 0.06 |
| hsa-miR-4722-5p | 100 | 0.87 | –1.13 | 0.75 | –1.1 | 0.03 | Robust | Blue | –0.01 |
| hsa-miR-4723-5p | 100 | 0.87 | –1.35 | 0.76 | –1.41 | 0.06 | Robust | Turquoise | –0.08 |
| hsa-miR-4725-3p | 100 | 0.93 | –1 | 0.88 | –1.03 | 0.03 | Robust | Blue | 0.01 |
| hsa-miR-4726-5p | 100 | 0.68 | –0.91 | 0.63 | –0.92 | 0.01 | Robust | Brown | 0.02 |
| hsa-miR-4728-5p | 100 | 0.93 | –1.11 | 0.92 | –1.09 | 0.02 | Robust | Pink |  |
| hsa-miR-4731-5p | 100 | 0.91 | –1.01 | 0.91 | –0.98 | 0.03 | Robust | Green |  |
| hsa-miR-4732-5p | 99.7 | 0.65 | –1.47 | 0.57 | –1.43 | 0.04 | Robust | Blue | –0.03 |
| hsa-miR-4736 | 100 | 0.77 | –1.1 | 0.68 | –1.03 | 0.07 | Robust | Blue | –0.08 |
| hsa-miR-4738-3p | 99.3 | 0.85 | –0.91 | 0.61 | –0.87 | 0.04 | Robust | Purple |  |
| hsa-miR-4739 | 100 | 0.93 | –0.97 | 0.91 | –0.98 | 0.01 | Robust | Green |  |
| hsa-miR-4741 | 100 | 0.95 | –1.03 | 0.9 | –1.01 | 0.02 | Robust | Turquoise | 0.03 |
| hsa-miR-4743-5p | 99.7 | 0.93 | –1.04 | 0.7 | –0.97 | 0.07 | Robust | Magenta | –0.07 |
| hsa-miR-4746-3p | 100 | 0.89 | –1.04 | 0.76 | –1.01 | 0.03 | Robust | Turquoise | –0.05 |
| hsa-miR-4747-5p | 99.3 | 0.85 | –1.26 | 0.81 | –1.22 | 0.04 | Robust | Red |  |
| hsa-miR-4749-5p | 100 | 0.95 | –1.01 | 0.87 | –1.08 | 0.07 | Robust | Turquoise | 0.1 |
| hsa-miR-4750-5p | 100 | 0.87 | –1.1 | 0.64 | –1.09 | 0.01 | Robust | Turquoise | 0.04 |
| hsa-miR-4751 | 99.3 | 0.83 | –0.98 | 0.7 | –0.98 | 0 | Robust | Purple |  |
| hsa-miR-4758-5p | 100 | 0.91 | –1.01 | 0.85 | –1.01 | 0 | Robust | Blue | 0.04 |
| hsa-miR-4763-3p | 100 | 0.92 | –0.95 | 0.78 | –1.02 | 0.07 | Robust | Turquoise | –0.03 |
| hsa-miR-4767 | 99.3 | 0.85 | –0.76 | 0.65 | –0.74 | 0.02 | Robust | Yellow | –0.02 |
| hsa-miR-4769-5p | 99.7 | 0.63 | –1.4 | 0.52 | –1.49 | 0.09 | Robust | Turquoise | 0 |
| hsa-miR-4783-3p | 100 | 0.9 | –1.05 | 0.78 | –1.02 | 0.03 | Robust | Turquoise | 0.09 |
| hsa-miR-4787-5p | 100 | 0.96 | –0.83 | 0.86 | –0.76 | 0.07 | Robust | Yellow | –0.11 |
| hsa-miR-4788 | 99.3 | 0.76 | –0.82 | 0.62 | –0.8 | 0.02 | Robust | Black |  |
| hsa-miR-4800-5p | 100 | 0.9 | –1.1 | 0.81 | –1.1 | 0 | Robust | Brown | 0.06 |
| hsa-miR-483-5p | 100 | 0.92 | –1.38 | 0.91 | –1.36 | 0.02 | Robust | Turquoise | –0.07 |
| hsa-miR-486-3p | 100 | 0.82 | –0.79 | 0.69 | –0.75 | 0.04 | Robust | Yellow | –0.02 |
| hsa-miR-491-5p | 100 | 0.55 | –1.03 | 0.6 | –1.08 | 0.05 | Robust | Brown | –0.01 |
| hsa-miR-498-5p | 100 | 0.81 | –0.85 | 0.68 | –0.82 | 0.03 | Robust | Black |  |
| hsa-miR-5001-5p | 100 | 0.8 | –1.05 | 0.64 | –1.11 | 0.06 | Robust | Turquoise | 0.04 |
| hsa-miR-5006-5p | 100 | 0.81 | –1 | 0.76 | –1.03 | 0.03 | Robust | Brown | –0.06 |
| hsa-miR-5008-5p | 100 | 0.88 | –0.99 | 0.73 | –0.96 | 0.03 | Robust | Yellow | 0.02 |
| hsa-miR-5010-5p | 100 | 0.57 | –0.94 | 0.62 | –0.99 | 0.05 | Robust | Brown | 0.01 |
| hsa-miR-504-3p | 100 | 0.85 | –1.14 | 0.72 | –1.16 | 0.02 | Robust | Turquoise | –0.02 |
| hsa-miR-5088-5p | 99.7 | 0.88 | –1.13 | 0.86 | –1.1 | 0.03 | Robust | Turquoise | –0.06 |
| hsa-miR-5090 | 100 | 0.89 | –0.97 | 0.8 | –0.91 | 0.06 | Robust | Black |  |
| hsa-miR-5189-5p | 100 | 0.82 | –1.13 | 0.7 | –1.16 | 0.03 | Robust | Turquoise | 0.05 |
| hsa-miR-5195-3p | 100 | 0.88 | –1.08 | 0.84 | –1.04 | 0.04 | Robust | Green |  |
| hsa-miR-5196-5p | 100 | 0.95 | –1.26 | 0.93 | –1.3 | 0.04 | Robust | Brown | 0.02 |
| hsa-miR-548q | 100 | 0.79 | –1.14 | 0.72 | –1.1 | 0.04 | Robust | Blue | 0.03 |
| hsa-miR-557 | 100 | 0.78 | –1 | 0.71 | –1 | 0 | Robust | Green |  |
| hsa-miR-5572 | 100 | 0.9 | –1.09 | 0.87 | –1.19 | 0.1 | Robust | Turquoise | –0.09 |
| hsa-miR-5587-3p | 99 | 0.83 | –1.13 | 0.79 | –1.18 | 0.05 | Robust | Red |  |
| hsa-miR-564 | 100 | 0.88 | –1 | 0.75 | –1.04 | 0.04 | Robust | Blue | 0.02 |
| hsa-miR-5698 | 100 | 0.87 | –1.12 | 0.72 | –1.06 | 0.06 | Robust | Pink |  |
| hsa-miR-5739 | 100 | 0.84 | –0.93 | 0.85 | –1 | 0.07 | Robust | Pink |  |
| hsa-miR-575 | 100 | 0.89 | –1.36 | 0.86 | –1.29 | 0.07 | Robust | Blue | 0.08 |
| hsa-miR-5787 | 100 | 0.94 | –0.92 | 0.96 | –0.96 | 0.04 | Robust | Blue | –0.04 |
| hsa-miR-602 | 100 | 0.87 | –0.85 | 0.7 | –0.85 | 0 | Robust | Yellow | –0.05 |
| hsa-miR-6068 | 100 | 0.77 | –1.17 | 0.59 | –1.22 | 0.05 | Robust | Turquoise | 0.06 |
| hsa-miR-6069 | 99 | 0.91 | –0.96 | 0.78 | –1 | 0.04 | Robust | Purple |  |
| hsa-miR-6075 | 100 | 0.86 | –0.95 | 0.66 | –1.01 | 0.06 | Robust | Turquoise | 0.04 |
| hsa-miR-6085 | 100 | 0.96 | –1.09 | 0.88 | –1.11 | 0.02 | Robust | Turquoise | –0.12 |
| hsa-miR-6086 | 100 | 0.91 | –1.12 | 0.82 | –1.08 | 0.04 | Robust | Pink |  |
| hsa-miR-6088 | 100 | 0.92 | –1.11 | 0.88 | –1.03 | 0.08 | Robust | Blue | –0.06 |
| hsa-miR-6089 | 100 | 0.96 | –0.86 | 0.89 | –0.81 | 0.05 | Robust | Gray |  |
| hsa-miR-6124 | 100 | 0.94 | –1.16 | 0.93 | –1.26 | 0.1 | Robust | Blue | –0.01 |
| hsa-miR-6125 | 100 | 0.95 | –0.93 | 0.9 | –0.96 | 0.03 | Robust | Yellow | –0.14 |
| hsa-miR-6126 | 100 | 0.95 | –1.02 | 0.92 | –1.04 | 0.02 | Robust | Pink |  |
| hsa-miR-6127 | 100 | 0.91 | –1.19 | 0.84 | –1.13 | 0.06 | Robust | Turquoise | 0.03 |
| hsa-miR-6132 | 100 | 0.79 | –1.14 | 0.62 | –1.19 | 0.05 | Robust | Turquoise | 0.02 |
| hsa-miR-6133 | 97.7 | 0.86 | –1.28 | 0.8 | –1.27 | 0.01 | Unrobust |  |  |
| hsa-miR-615-5p | 100 | 0.82 | –0.95 | 0.61 | –0.99 | 0.04 | Robust | Turquoise | 0 |
| hsa-miR-6165 | 100 | 0.72 | –1.13 | 0.67 | –1.18 | 0.05 | Robust | Blue | –0.03 |
| hsa-miR-637 | 99.3 | 0.66 | –0.91 | 0.63 | –0.87 | 0.04 | Robust | Yellow | 0.03 |
| hsa-miR-638 | 100 | 0.95 | –0.88 | 0.84 | –0.91 | 0.03 | Robust | Yellow | –0.15 |
| hsa-miR-642b-3p | 100 | 0.76 | –0.84 | 0.62 | –0.79 | 0.05 | Robust | Black |  |
| hsa-miR-6510-5p | 100 | 0.85 | –1.18 | 0.87 | –1.27 | 0.09 | Robust | Pink |  |
| hsa-miR-6511a-5p | 100 | 0.72 | –1.11 | 0.52 | –1.21 | 0.1 | Robust | Turquoise | 0.04 |
| hsa-miR-6511b-5p | 100 | 0.71 | –1.15 | 0.53 | –1.15 | 0 | Robust | Turquoise | 0.05 |
| hsa-miR-652-5p | 100 | 0.76 | –1.03 | 0.73 | –1.05 | 0.02 | Robust | Brown | 0.03 |
| hsa-miR-6529-5p | 98.7 | 0.71 | –1.02 | 0.53 | –0.98 | 0.04 | Unrobust |  |  |
| hsa-miR-658 | 100 | 0.75 | –1 | 0.75 | –1.09 | 0.09 | Robust | Brown | –0.02 |
| hsa-miR-663a | 100 | 0.92 | –0.98 | 0.75 | –1.03 | 0.05 | Robust | Turquoise | 0.05 |
| hsa-miR-671-5p | 100 | 0.63 | –1.02 | 0.64 | –0.97 | 0.05 | Robust | Brown | 0.01 |
| hsa-miR-6716-5p | 100 | 0.95 | –1.16 | 0.9 | –1.18 | 0.02 | Robust | Turquoise | –0.02 |
| hsa-miR-6721-5p | 100 | 0.88 | –1.12 | 0.82 | –1.1 | 0.02 | Robust | Turquoise | 0.02 |
| hsa-miR-6722-3p | 100 | 0.88 | –1.07 | 0.8 | –1.06 | 0.01 | Robust | Blue | –0.01 |
| hsa-miR-6722-5p | 100 | 0.85 | –1 | 0.67 | –0.95 | 0.05 | Robust | Blue | –0.01 |
| hsa-miR-6724-5p | 100 | 0.83 | –0.9 | 0.76 | –1 | 0.1 | Robust | Brown | 0.02 |
| hsa-miR-6726-5p | 100 | 0.83 | –1.06 | 0.77 | –1.1 | 0.04 | Robust | Blue | –0.02 |
| hsa-miR-6727-3p | 97 | 0.74 | –0.73 | 0.64 | –0.81 | 0.08 | Unrobust |  |  |
| hsa-miR-6727-5p | 100 | 0.91 | –0.88 | 0.88 | –0.88 | 0 | Robust | Yellow | –0.08 |
| hsa-miR-6729-5p | 100 | 0.94 | –0.89 | 0.91 | –0.9 | 0.01 | Robust | Yellow | –0.13 |
| hsa-miR-6732-5p | 100 | 0.92 | –0.94 | 0.81 | –0.96 | 0.02 | Robust | Turquoise | –0.01 |
| hsa-miR-6735-5p | 100 | 0.9 | –1.16 | 0.84 | –1.09 | 0.07 | Robust | Turquoise | 0.01 |
| hsa-miR-6737-5p | 100 | 0.87 | –1 | 0.82 | –1 | 0 | Robust | Brown | 0.03 |
| hsa-miR-6738-5p | 100 | 0.9 | –1.02 | 0.87 | –0.99 | 0.03 | Robust | Black |  |
| hsa-miR-6743-5p | 100 | 0.91 | –0.98 | 0.87 | –0.95 | 0.03 | Robust | Turquoise | –0.06 |
| hsa-miR-6746-5p | 100 | 0.87 | –1.04 | 0.85 | –1.09 | 0.05 | Robust | Brown | 0.07 |
| hsa-miR-6749-5p | 100 | 0.9 | –0.93 | 0.89 | –0.9 | 0.03 | Robust | Yellow | 0.01 |
| hsa-miR-6751-5p | 100 | 0.88 | –1.1 | 0.66 | –1.16 | 0.06 | Robust | Brown | 0.04 |
| hsa-miR-6752-5p | 100 | 0.93 | –0.89 | 0.94 | –0.93 | 0.04 | Robust | Blue | –0.07 |
| hsa-miR-6753-5p | 100 | 0.79 | –1.05 | 0.54 | –1.08 | 0.03 | Robust | Turquoise | 0.06 |
| hsa-miR-6754-5p | 100 | 0.85 | –0.98 | 0.68 | –1 | 0.02 | Robust | Magenta | –0.06 |
| hsa-miR-675-5p | 100 | 0.87 | –1.1 | 0.86 | –1.17 | 0.07 | Robust | Brown | 0 |
| hsa-miR-6756-5p | 100 | 0.93 | –1.07 | 0.79 | –1.1 | 0.03 | Robust | Turquoise | 0.08 |
| hsa-miR-6757-5p | 100 | 0.61 | –0.93 | 0.72 | –0.97 | 0.04 | Robust | Brown | –0.02 |
| hsa-miR-6762-5p | 100 | 0.87 | –0.99 | 0.85 | –0.91 | 0.08 | Robust | Black |  |
| hsa-miR-6763-3p | 98.7 | 0.79 | –0.72 | 0.8 | –0.78 | 0.06 | Unrobust |  |  |
| hsa-miR-6763-5p | 100 | 0.88 | –1.21 | 0.82 | –1.16 | 0.05 | Robust | Blue | 0.05 |
| hsa-miR-6765-5p | 100 | 0.98 | –1 | 0.96 | –0.98 | 0.02 | Robust | Yellow | 0.11 |
| hsa-miR-6766-5p | 99.7 | 0.91 | –1.1 | 0.86 | –1.14 | 0.04 | Robust | Turquoise | 0.09 |
| hsa-miR-6769a-5p | 100 | 0.89 | –1.02 | 0.77 | –1.04 | 0.02 | Robust | Brown | –0.01 |
| hsa-miR-6769b-5p | 100 | 0.84 | –1.18 | 0.83 | –1.18 | 0 | Robust | Brown | –0.03 |
| hsa-miR-6770-3p | 99.7 | 0.66 | –0.76 | 0.69 | –0.85 | 0.09 | Robust | Red |  |
| hsa-miR-6771-5p | 100 | 0.92 | –0.89 | 0.87 | –0.88 | 0.01 | Robust | Blue | 0.01 |
| hsa-miR-6772-5p | 99 | 0.74 | –1.11 | 0.64 | –1.06 | 0.05 | Robust | Red |  |
| hsa-miR-6774-5p | 100 | 0.86 | –0.89 | 0.86 | –0.93 | 0.04 | Robust | Green |  |
| hsa-miR-6775-5p | 100 | 0.93 | –1.09 | 0.85 | –1.09 | 0 | Robust | Turquoise | 0.1 |
| hsa-miR-6776-5p | 100 | 0.89 | –1.12 | 0.87 | –1.15 | 0.03 | Robust | Turquoise | –0.08 |
| hsa-miR-6777-5p | 100 | 0.8 | –1.12 | 0.8 | –1.14 | 0.02 | Robust | Blue | 0.01 |
| hsa-miR-6778-5p | 100 | 0.7 | –1.15 | 0.54 | –1.14 | 0.01 | Robust | Blue | –0.05 |
| hsa-miR-6779-5p | 100 | 0.87 | –1.2 | 0.81 | –1.14 | 0.06 | Robust | Blue | 0.02 |
| hsa-miR-6781-5p | 100 | 0.96 | –0.94 | 0.88 | –0.96 | 0.02 | Robust | Turquoise | 0.04 |
| hsa-miR-6784-5p | 100 | 0.84 | –0.86 | 0.75 | –0.81 | 0.05 | Robust | Yellow | –0.01 |
| hsa-miR-6785-5p | 100 | 0.77 | –0.79 | 0.76 | –0.7 | 0.09 | Robust | Black |  |
| hsa-miR-6786-5p | 100 | 0.96 | –0.92 | 0.96 | –0.91 | 0.01 | Robust | Yellow | –0.01 |
| hsa-miR-6789-5p | 100 | 0.91 | –0.81 | 0.83 | –0.78 | 0.03 | Robust | Yellow | –0.06 |
| hsa-miR-6790-3p | 99.3 | 0.84 | –0.93 | 0.63 | –0.95 | 0.02 | Robust | Purple |  |
| hsa-miR-6790-5p | 99.3 | 0.86 | –1.02 | 0.82 | –1.1 | 0.08 | Robust | Red |  |
| hsa-miR-6791-5p | 100 | 0.9 | –0.95 | 0.79 | –0.96 | 0.01 | Robust | Turquoise | –0.04 |
| hsa-miR-6792-5p | 100 | 0.73 | –1.11 | 0.53 | –1.13 | 0.02 | Robust | Turquoise | 0.04 |
| hsa-miR-6794-5p | 100 | 0.92 | –1.08 | 0.86 | –1.04 | 0.04 | Robust | Turquoise | 0.02 |
| hsa-miR-6795-5p | 100 | 0.94 | –1.15 | 0.92 | –1.11 | 0.04 | Robust | Green |  |
| hsa-miR-6797-5p | 100 | 0.93 | –1.29 | 0.88 | –1.26 | 0.03 | Robust | Turquoise | –0.08 |
| hsa-miR-6798-5p | 100 | 0.91 | –0.98 | 0.94 | –1.04 | 0.06 | Robust | Blue | –0.11 |
| hsa-miR-6799-5p | 100 | 0.92 | –1.24 | 0.75 | –1.31 | 0.07 | Robust | Turquoise | 0.09 |
| hsa-miR-6800-5p | 100 | 0.95 | –0.97 | 0.95 | –0.95 | 0.02 | Robust | Yellow | 0.03 |
| hsa-miR-6802-5p | 100 | 0.85 | –1.05 | 0.92 | –1.1 | 0.05 | Robust | Brown | –0.01 |
|  |  |  |  |  |  |  |  |  |  |
| hsa-miR-6804-3p | 99 | 0.71 | –0.71 | 0.57 | –0.73 | 0.02 | Robust | Red |  |
| hsa-miR-6805-5p | 100 | 0.97 | –1.03 | 0.97 | –1 | 0.03 | Robust | Yellow | –0.06 |
| hsa-miR-6806-5p | 100 | 0.88 | –0.92 | 0.72 | –0.91 | 0.01 | Robust | Yellow | –0.06 |
| hsa-miR-6807-5p | 100 | 0.67 | –0.99 | 0.69 | –0.99 | 0 | Robust | Brown | 0.01 |
| hsa-miR-6810-5p | 100 | 0.84 | –1.17 | 0.82 | –1.14 | 0.03 | Robust | Blue | –0.01 |
| hsa-miR-6812-5p | 100 | 0.92 | –1.23 | 0.87 | –1.22 | 0.01 | Robust | Brown | –0.06 |
| hsa-miR-6813-5p | 100 | 0.82 | –1.18 | 0.72 | –1.13 | 0.05 | Robust | Blue | –0.01 |
| hsa-miR-6816-5p | 100 | 0.95 | –0.94 | 0.89 | –0.97 | 0.03 | Robust | Turquoise | –0.04 |
| hsa-miR-6820-5p | 100 | 0.8 | –1.13 | 0.72 | –1.15 | 0.02 | Robust | Blue | 0.02 |
| hsa-miR-6821-5p | 100 | 0.94 | –1.15 | 0.85 | –1.17 | 0.02 | Robust | Blue | 0.06 |
| hsa-miR-6824-5p | 100 | 0.76 | –1.03 | 0.77 | –1.1 | 0.07 | Robust | Brown | 0.04 |
| hsa-miR-6825-3p | 99.7 | 0.87 | –0.86 | 0.82 | –0.89 | 0.03 | Robust | Blue | –0.08 |
| hsa-miR-6827-5p | 99.7 | 0.88 | –1.08 | 0.68 | –1.09 | 0.01 | Robust | Purple |  |
| hsa-miR-6829-5p | 100 | 0.84 | –1.18 | 0.65 | –1.2 | 0.02 | Robust | Turquoise | 0.04 |
| hsa-miR-6830-5p | 99.3 | 0.79 | –1.05 | 0.64 | –1.08 | 0.03 | Robust | Red |  |
| hsa-miR-6831-5p | 99.7 | 0.84 | –1.27 | 0.8 | –1.3 | 0.03 | Robust | Brown | 0.03 |
| hsa-miR-6835-5p | 98.7 | 0.71 | –1.18 | 0.71 | –1.13 | 0.05 | Unrobust |  |  |
| hsa-miR-6837-5p | 99 | 0.86 | –0.96 | 0.67 | –1.04 | 0.08 | Robust | Red |  |
| hsa-miR-6840-3p | 100 | 0.56 | –0.66 | 0.56 | –0.71 | 0.05 | Robust | Turquoise | –0.02 |
| hsa-miR-6845-3p | 99 | 0.76 | –0.72 | 0.73 | –0.65 | 0.07 | Robust | Red |  |
| hsa-miR-6845-5p | 100 | 0.91 | –0.95 | 0.92 | –0.91 | 0.04 | Robust | Black |  |
| hsa-miR-6846-5p | 99.7 | 0.86 | –1.18 | 0.79 | –1.12 | 0.06 | Robust | Blue | 0.02 |
| hsa-miR-6848-5p | 100 | 0.87 | –1.2 | 0.77 | –1.18 | 0.02 | Robust | Blue | 0.01 |
| hsa-miR-6850-5p | 100 | 0.96 | –0.86 | 0.93 | –0.87 | 0.01 | Robust | Yellow | –0.06 |
| hsa-miR-6851-5p | 100 | 0.83 | –1.05 | 0.63 | –1 | 0.05 | Robust | Blue | 0 |
| hsa-miR-6858-5p | 100 | 0.91 | –0.99 | 0.76 | –1.01 | 0.02 | Robust | Turquoise | 0.08 |
| hsa-miR-6860 | 100 | 0.89 | –1.13 | 0.78 | –1.16 | 0.03 | Robust | Turquoise | 0.07 |
| hsa-miR-6861-5p | 100 | 0.73 | –1.18 | 0.6 | –1.12 | 0.06 | Robust | Blue | 0.01 |
| hsa-miR-6865-5p | 99.7 | 0.87 | –1.13 | 0.77 | –1.06 | 0.07 | Robust | Blue | –0.03 |
| hsa-miR-6871-5p | 99 | 0.8 | –0.98 | 0.67 | –0.98 | 0 | Robust | Blue | –0.01 |
| hsa-miR-6875-5p | 100 | 0.77 | –0.94 | 0.65 | –0.87 | 0.07 | Robust | Black |  |
| hsa-miR-6879-3p | 99.3 | 0.75 | –0.72 | 0.67 | –0.75 | 0.03 | Robust | Yellow | –0.03 |
| hsa-miR-6879-5p | 100 | 0.93 | –1.09 | 0.94 | –1.16 | 0.07 | Robust | Blue | –0.04 |
| hsa-miR-6880-3p | 99.3 | 0.61 | –0.58 | 0.53 | –0.66 | 0.08 | Robust | Red |  |
| hsa-miR-6880-5p | 100 | 0.81 | –1.01 | 0.69 | –1.05 | 0.04 | Robust | Green |  |
| hsa-miR-6886-3p | 99 | 0.72 | –0.84 | 0.65 | –0.79 | 0.05 | Robust | Red |  |
| hsa-miR-6887-5p | 100 | 0.93 | –1.15 | 0.91 | –1.12 | 0.03 | Robust | Green |  |
| hsa-miR-6891-5p | 100 | 0.91 | –1.15 | 0.89 | –1.21 | 0.06 | Robust | Blue | –0.03 |
| hsa-miR-6892-5p | 98.7 | 0.76 | –0.9 | 0.68 | –0.95 | 0.05 | Unrobust |  |  |
| hsa-miR-6893-5p | 100 | 0.82 | –1.06 | 0.67 | –1.02 | 0.04 | Robust | Blue | 0.03 |
| hsa-miR-6894-5p | 100 | 0.92 | –1.14 | 0.76 | –1.09 | 0.05 | Robust | Magenta | –0.09 |
| hsa-miR-7106-5p | 100 | 0.93 | –1.26 | 0.93 | –1.29 | 0.03 | Robust | Brown | –0.06 |
| hsa-miR-7107-5p | 100 | 0.94 | –1.16 | 0.9 | –1.25 | 0.09 | Robust | Brown | –0.04 |
| hsa-miR-7108-5p | 100 | 0.91 | –0.98 | 0.77 | –0.98 | 0 | Robust | Turquoise | 0.02 |
| hsa-miR-7109-5p | 100 | 0.9 | –1.12 | 0.9 | –1.09 | 0.03 | Robust | Brown | 0.1 |
| hsa-miR-711 | 100 | 0.8 | –0.85 | 0.73 | –0.9 | 0.05 | Robust | Pink |  |
| hsa-miR-7110-5p | 100 | 0.91 | –1.23 | 0.83 | –1.28 | 0.05 | Robust | Turquoise | –0.11 |
| hsa-miR-7111-5p | 100 | 0.95 | –1.23 | 0.93 | –1.27 | 0.04 | Robust | Brown | 0.06 |
| hsa-miR-7112-5p | 99.7 | 0.85 | –0.98 | 0.74 | –1.08 | 0.1 | Robust | Turquoise | 0.03 |
| hsa-miR-7155-5p | 99.7 | 0.91 | –1.02 | 0.78 | –1.04 | 0.02 | Robust | Purple |  |
| hsa-miR-7160-5p | 99 | 0.82 | –0.91 | 0.58 | –0.96 | 0.05 | Robust | Red |  |
| hsa-miR-718 | 100 | 0.87 | –0.76 | 0.78 | –0.84 | 0.08 | Robust | Green |  |
| hsa-miR-760 | 100 | 0.91 | –0.94 | 0.91 | –0.96 | 0.02 | Robust | Brown | –0.03 |
| hsa-miR-762 | 100 | 0.9 | –0.86 | 0.81 | –0.81 | 0.05 | Robust | Yellow | –0.01 |
| hsa-miR-765 | 100 | 0.57 | –1.09 | 0.59 | –1.05 | 0.04 | Robust | Brown | –0.02 |
| hsa-miR-7845-5p | 100 | 0.95 | –1.15 | 0.96 | –1.16 | 0.01 | Robust | Brown | 0 |
| hsa-miR-7846-3p | 100 | 0.81 | –0.94 | 0.75 | –1 | 0.06 | Robust | Pink |  |
| hsa-miR-7847-3p | 100 | 0.95 | –1.1 | 0.88 | –1.06 | 0.04 | Robust | Magenta | –0.13 |
| hsa-miR-8052 | 100 | 0.9 | –0.99 | 0.77 | –0.97 | 0.02 | Robust | Turquoise | 0.13 |
| hsa-miR-8059 | 100 | 0.8 | –0.93 | 0.82 | –0.96 | 0.03 | Robust | Green |  |
| hsa-miR-8063 | 100 | 0.84 | –0.97 | 0.77 | –0.92 | 0.05 | Robust | Blue | 0.02 |
| hsa-miR-8064 | 99.3 | 0.77 | –1.01 | 0.59 | –1.08 | 0.07 | Robust | Red |  |
| hsa-miR-8071 | 100 | 0.87 | –1.14 | 0.66 | –1.07 | 0.07 | Robust | Brown | 0.04 |
| hsa-miR-8072 | 100 | 0.97 | –0.92 | 0.93 | –0.93 | 0.01 | Robust | Turquoise | –0.1 |
| hsa-miR-877-5p | 99.7 | 0.85 | –1.09 | 0.82 | –1.11 | 0.02 | Robust | Purple |  |
| hsa-miR-920 | 99.3 | 0.86 | –0.99 | 0.75 | –0.93 | 0.06 | Robust | Purple |  |
| hsa-miR-935 | 99 | 0.75 | –0.7 | 0.68 | –0.74 | 0.04 | Robust | Red |  |
| hsa-miR-937-5p | 100 | 0.95 | –1.17 | 0.88 | –1.2 | 0.03 | Robust | Turquoise | 0.04 |
| hsa-miR-939-5p | 100 | 0.88 | –1.15 | 0.84 | –1.2 | 0.05 | Robust | Turquoise | –0.12 |
| hsa-miR-9899 | 100 | 0.77 | –0.66 | 0.71 | –0.68 | 0.02 | Robust | Black |  |
| hsa-miR-10396a-3p | 100 | 0.6 | –0.77 | 0.41 | –0.68 | 0.09 | Unrobust |  |  |
| hsa-miR-10400-5p | 100 | 0.9 | –0.85 | 0 | 0 | 0.85 | Unrobust |  |  |
| hsa-miR-1185-2-3p | 99.7 | 0.73 | –1 | 0.78 | –1.12 | 0.12 | Unrobust |  |  |
| hsa-miR-12116 | 96.7 | 0.58 | –0.62 | 0.67 | –0.83 | 0.21 | Unrobust |  |  |
| hsa-miR-12117 | 94.3 | 0.55 | –0.6 | 0.22 | –0.66 | 0.06 | Unrobust |  |  |
| hsa-miR-12121 | 100 | 0.73 | –1.24 | 0.71 | –1.09 | 0.15 | Unrobust |  |  |
| hsa-miR-1224-5p | 99 | 0.85 | –0.99 | 0.7 | –0.86 | 0.13 | Unrobust |  |  |
| hsa-miR-1225-3p | 100 | 0.66 | –0.73 | 0.48 | –0.8 | 0.07 | Unrobust |  |  |
| hsa-miR-1229-5p | 100 | 0.92 | –1.06 | 0.94 | –1.21 | 0.15 | Unrobust |  |  |
| hsa-miR-1238-5p | 100 | 0.74 | –0.74 | 0.81 | –0.88 | 0.14 | Unrobust |  |  |
| hsa-miR-125a-3p | 100 | 0.79 | –1.62 | 0.53 | –1.12 | 0.5 | Unrobust |  |  |
| hsa-miR-125b-1-3p | 100 | 0.53 | –1.62 | 0.52 | –1.16 | 0.46 | Unrobust |  |  |
| hsa-miR-1260a | 100 | 0.51 | –0.75 | 0.23 | –0.65 | 0.1 | Unrobust |  |  |
| hsa-miR-1260b | 100 | 0.51 | –0.81 | 0.3 | –0.78 | 0.03 | Unrobust |  |  |
| hsa-miR-1273c | 100 | 0.56 | –0.92 | 0.46 | –0.98 | 0.06 | Unrobust |  |  |
| hsa-miR-1275 | 100 | 0.91 | –1.08 | 0.93 | –1.22 | 0.14 | Unrobust |  |  |
| hsa-miR-1281 | 100 | 0.64 | –0.61 | 0.41 | –0.67 | 0.06 | Unrobust |  |  |
| hsa-miR-128-2-5p | 100 | 0.68 | –0.63 | 0.47 | –0.61 | 0.02 | Unrobust |  |  |
| hsa-miR-134-3p | 94 | 0.61 | –0.89 | 0.33 | –0.89 | 0 | Unrobust |  |  |
| hsa-miR-150-3p | 100 | 0.79 | –1.1 | 0.47 | –0.92 | 0.18 | Unrobust |  |  |
| hsa-miR-185-3p | 97 | 0.72 | –1.09 | 0.63 | –0.97 | 0.12 | Unrobust |  |  |
| hsa-miR-193a-5p | 98.7 | 0.71 | –0.96 | 0.59 | –1.16 | 0.2 | Unrobust |  |  |
| hsa-miR-193b-5p | 100 | 0.86 | –1.11 | 0.78 | –1.23 | 0.12 | Unrobust |  |  |
| hsa-miR-204-3p | 100 | 0.75 | –0.99 | 0.37 | –0.72 | 0.27 | Unrobust |  |  |
| hsa-miR-210-3p | 96.7 | 0.52 | –0.89 | 0.36 | –1.1 | 0.21 | Unrobust |  |  |
| hsa-miR-2276-3p | 97.7 | 0.7 | –0.77 | 0.4 | –0.89 | 0.12 | Unrobust |  |  |
| hsa-miR-2392 | 100 | 0.74 | –1.27 | 0.42 | –1.34 | 0.07 | Unrobust |  |  |
| hsa-miR-23a-5p | 98 | 0.68 | –0.87 | 0.53 | –1.03 | 0.16 | Unrobust |  |  |
| hsa-miR-296-5p | 98 | 0.55 | –0.62 | 0.67 | –0.91 | 0.29 | Unrobust |  |  |
| hsa-miR-3059-3p | 94.7 | 0.67 | –1.2 | 0.49 | –1.37 | 0.17 | Unrobust |  |  |
| hsa-miR-3132 | 97.3 | 0.75 | –1.1 | 0.68 | –1.31 | 0.21 | Unrobust |  |  |
| hsa-miR-3138 | 94.7 | 0.7 | –0.95 | 0.56 | –1.08 | 0.13 | Unrobust |  |  |
| hsa-miR-3141 | 100 | 0.96 | –1.02 | 0.95 | –1.15 | 0.13 | Unrobust |  |  |
| hsa-miR-3144-5p | 96 | 0.67 | –0.84 | 0.64 | –1.09 | 0.25 | Unrobust |  |  |
| hsa-miR-3150b-5p | 96 | 0.56 | –0.71 | 0.44 | –0.98 | 0.27 | Unrobust |  |  |
| hsa-miR-3153 | 95.7 | 0.79 | –0.92 | 0.39 | –0.91 | 0.01 | Unrobust |  |  |
| hsa-miR-3158-5p | 99 | 0.54 | –0.74 | 0.44 | –0.82 | 0.08 | Unrobust |  |  |
| hsa-miR-3175 | 100 | 0.73 | –1.01 | 0.8 | –1.16 | 0.15 | Unrobust |  |  |
| hsa-miR-3176 | 99.3 | 0.77 | –0.87 | 0.43 | –0.69 | 0.18 | Unrobust |  |  |
| hsa-miR-3177-5p | 99 | 0.7 | –0.71 | 0.28 | –0.64 | 0.07 | Unrobust |  |  |
| hsa-miR-3202 | 95.3 | 0.69 | –1.03 | 0.69 | –1.32 | 0.29 | Unrobust |  |  |
| hsa-miR-320c | 95 | 0.62 | –0.98 | 0.73 | –1.28 | 0.3 | Unrobust |  |  |
| hsa-miR-323a-5p | 98.7 | 0.56 | –0.83 | 0.41 | –0.74 | 0.09 | Unrobust |  |  |
| hsa-miR-3616-3p | 100 | 0.64 | –0.83 | 0.44 | –0.79 | 0.04 | Unrobust |  |  |
| hsa-miR-3619-3p | 100 | 0.81 | –1.15 | 0.45 | –0.92 | 0.23 | Unrobust |  |  |
| hsa-miR-3622b-5p | 100 | 0.77 | –1.13 | 0.38 | –1.24 | 0.11 | Unrobust |  |  |
| hsa-miR-3648 | 100 | 0.53 | –0.72 | 0.48 | –0.74 | 0.02 | Unrobust |  |  |
| hsa-miR-365a-5p | 100 | 0.71 | –0.92 | 0.39 | –0.95 | 0.03 | Unrobust |  |  |
| hsa-miR-3663-3p | 100 | 0.67 | –0.59 | 0.42 | –0.61 | 0.02 | Unrobust |  |  |
| hsa-miR-3678-3p | 99.3 | 0.79 | –1.18 | 0.54 | –1.07 | 0.11 | Unrobust |  |  |
| hsa-miR-3679-5p | 100 | 0.83 | –0.98 | 0.88 | –1.09 | 0.11 | Unrobust |  |  |
| hsa-miR-3682-3p | 98 | 0.53 | –0.81 | 0.33 | –0.9 | 0.09 | Unrobust |  |  |
| hsa-miR-3713 | 93.7 | 0.66 | –0.82 | 0.13 | –0.5 | 0.32 | Unrobust |  |  |
| hsa-miR-373-5p | 98.3 | 0.57 | –0.65 | 0.63 | –0.8 | 0.15 | Unrobust |  |  |
| hsa-miR-375-5p | 100 | 0.65 | –0.95 | 0.34 | –0.7 | 0.25 | Unrobust |  |  |
| hsa-miR-378f | 94 | 0.61 | –1.01 | 0.58 | –1.18 | 0.17 | Unrobust |  |  |
| hsa-miR-3918 | 100 | 0.74 | –1.03 | 0.47 | –0.9 | 0.13 | Unrobust |  |  |
| hsa-miR-3944-3p | 92.7 | 0.6 | –0.77 | 0.62 | –0.97 | 0.2 | Unrobust |  |  |
| hsa-miR-3945 | 97.7 | 0.78 | –1.13 | 0.83 | –1.29 | 0.16 | Unrobust |  |  |
| hsa-miR-3960 | 100 | 0.93 | –0.83 | 0 | 0 | 0.83 | Unrobust |  |  |
| hsa-miR-423-5p | 100 | 0.87 | –1.07 | 0.65 | –0.94 | 0.13 | Unrobust |  |  |
| hsa-miR-4257 | 100 | 0.9 | –1.07 | 0.62 | –1.18 | 0.11 | Unrobust |  |  |
| hsa-miR-4269 | 99.3 | 0.77 | –0.91 | 0.46 | –0.79 | 0.12 | Unrobust |  |  |
| hsa-miR-4294 | 100 | 0.64 | –0.92 | 0.62 | –1.07 | 0.15 | Unrobust |  |  |
| hsa-miR-4314 | 98 | 0.64 | –0.88 | 0.21 | –0.66 | 0.22 | Unrobust |  |  |
| hsa-miR-4429 | 100 | 0.66 | –1.15 | 0.48 | –1.26 | 0.11 | Unrobust |  |  |
| hsa-miR-4440 | 94.7 | 0.65 | –0.74 | 0.48 | –0.96 | 0.22 | Unrobust |  |  |
| hsa-miR-4450 | 100 | 0.84 | –1.21 | 0.59 | –1.01 | 0.2 | Unrobust |  |  |
| hsa-miR-4470 | 91 | 0.57 | –0.83 | 0.39 | –0.97 | 0.14 | Unrobust |  |  |
| hsa-miR-4476 | 100 | 0.8 | –1.13 | 0.5 | –0.96 | 0.17 | Unrobust |  |  |
| hsa-miR-4481 | 100 | 0.84 | –1.2 | 0.59 | –0.99 | 0.21 | Unrobust |  |  |
| hsa-miR-4484 | 100 | 0.8 | –0.88 | 0.76 | –0.99 | 0.11 | Unrobust |  |  |
| hsa-miR-4489 | 97 | 0.73 | –0.96 | 0.62 | –1.09 | 0.13 | Unrobust |  |  |
| hsa-miR-4496 | 99.7 | 0.69 | –0.87 | 0.64 | –1 | 0.13 | Unrobust |  |  |
| hsa-miR-4497 | 100 | 0.73 | –0.89 | 0.4 | –0.81 | 0.08 | Unrobust |  |  |
| hsa-miR-4499 | 98.3 | 0.78 | –1.03 | 0.76 | –1.24 | 0.21 | Unrobust |  |  |
| hsa-miR-4644 | 99 | 0.62 | –0.8 | 0.52 | –0.99 | 0.19 | Unrobust |  |  |
| hsa-miR-4646-5p | 100 | 0.38 | –0.87 | 0.52 | –0.94 | 0.07 | Unrobust |  |  |
| hsa-miR-4649-5p | 100 | 0.51 | –0.53 | 0.27 | –0.44 | 0.09 | Unrobust |  |  |
| hsa-miR-4655-5p | 100 | 0.68 | –0.86 | 0.81 | –1 | 0.14 | Unrobust |  |  |
| hsa-miR-4684-3p | 98.3 | 0.76 | –1.19 | 0.31 | –1.05 | 0.14 | Unrobust |  |  |
| hsa-miR-4685-5p | 99.3 | 0.84 | –0.97 | 0.65 | –0.84 | 0.13 | Unrobust |  |  |
| hsa-miR-4688 | 100 | 0.76 | –0.78 | 0.85 | –0.92 | 0.14 | Unrobust |  |  |
| hsa-miR-4695-3p | 99 | 0.62 | –0.86 | 0.42 | –0.81 | 0.05 | Unrobust |  |  |
| hsa-miR-4708-3p | 97.7 | 0.51 | –0.93 | 0.34 | –1.12 | 0.19 | Unrobust |  |  |
| hsa-miR-4716-3p | 96 | 0.79 | –1.27 | 0.79 | –1.47 | 0.2 | Unrobust |  |  |
| hsa-miR-4726-3p | 99 | 0.57 | –0.62 | 0.49 | –0.67 | 0.05 | Unrobust |  |  |
| hsa-miR-4730 | 100 | 0.71 | –0.92 | 0.28 | –0.7 | 0.22 | Unrobust |  |  |
| hsa-miR-4740-3p | 98 | 0.89 | –1.19 | 0.56 | –1.03 | 0.16 | Unrobust |  |  |
| hsa-miR-4745-5p | 100 | 0.72 | –0.76 | 0.39 | –0.7 | 0.06 | Unrobust |  |  |
| hsa-miR-4748 | 98.7 | 0.75 | –0.94 | 0.67 | –1.06 | 0.12 | Unrobust |  |  |
| hsa-miR-4749-3p | 98.7 | 0.62 | –0.61 | 0.65 | –0.74 | 0.13 | Unrobust |  |  |
| hsa-miR-4756-5p | 100 | 0.51 | –1.05 | 0.54 | –0.91 | 0.14 | Unrobust |  |  |
| hsa-miR-4758-3p | 99.3 | 0.65 | –0.67 | 0.43 | –0.59 | 0.08 | Unrobust |  |  |
| hsa-miR-4763-5p | 97 | 0.57 | –0.56 | 0.67 | –0.86 | 0.3 | Unrobust |  |  |
| hsa-miR-4776-5p | 99.7 | 0.64 | –1.11 | 0.47 | –1.17 | 0.06 | Unrobust |  |  |
| hsa-miR-493-3p | 96 | 0.73 | –1.04 | 0.49 | –1.01 | 0.03 | Unrobust |  |  |
| hsa-miR-494-3p | 100 | 0.52 | –1.13 | 0.49 | –1 | 0.13 | Unrobust |  |  |
| hsa-miR-5100 | 100 | 0.48 | –1.15 | 0.47 | –1.04 | 0.11 | Unrobust |  |  |
| hsa-miR-513a-5p | 99.3 | 0.51 | –0.99 | 0.26 | –1.14 | 0.15 | Unrobust |  |  |
| hsa-miR-513b-5p | 94.7 | 0.66 | –0.88 | 0.46 | –1.06 | 0.18 | Unrobust |  |  |
| hsa-miR-513c-5p | 97 | 0.6 | –0.84 | 0.35 | –0.94 | 0.1 | Unrobust |  |  |
| hsa-miR-514b-5p | 98.7 | 0.59 | –0.73 | 0.64 | –0.99 | 0.26 | Unrobust |  |  |
| hsa-miR-542-5p | 96.3 | 0.52 | –0.73 | 0.11 | –0.39 | 0.34 | Unrobust |  |  |
| hsa-miR-550a-3-5p | 95.7 | 0.65 | –0.82 | 0.69 | –1.18 | 0.36 | Unrobust |  |  |
| hsa-miR-550a-5p | 100 | 0.7 | –1.03 | 0.38 | –0.91 | 0.12 | Unrobust |  |  |
| hsa-miR-5585-3p | 100 | 0.62 | –1.04 | 0.41 | –1.02 | 0.02 | Unrobust |  |  |
| hsa-miR-5589-5p | 99 | 0.58 | –1.07 | 0.57 | –1.18 | 0.11 | Unrobust |  |  |
| hsa-miR-572 | 100 | 0.73 | –1.07 | 0.55 | –1.22 | 0.15 | Unrobust |  |  |
| hsa-miR-6081 | 96.7 | 0.71 | –0.72 | 0.42 | –0.87 | 0.15 | Unrobust |  |  |
| hsa-miR-6090 | 100 | 0.97 | –0.93 | 0.9 | –0.82 | 0.11 | Unrobust |  |  |
| hsa-miR-612 | 97.3 | 0.69 | –0.78 | 0.82 | –1.07 | 0.29 | Unrobust |  |  |
| hsa-miR-6129 | 99 | 0.78 | –1.14 | 0.53 | –1.29 | 0.15 | Unrobust |  |  |
| hsa-miR-614 | 99.7 | 0.77 | –1.22 | 0.46 | –1.01 | 0.21 | Unrobust |  |  |
| hsa-miR-619-5p | 100 | 0.52 | –0.95 | 0.42 | –1.01 | 0.06 | Unrobust |  |  |
| hsa-miR-650 | 99 | 0.72 | –0.93 | 0.72 | –1.12 | 0.19 | Unrobust |  |  |
| hsa-miR-659-3p | 98.7 | 0.74 | –0.85 | 0.66 | –0.96 | 0.11 | Unrobust |  |  |
| hsa-miR-6717-5p | 94.3 | 0.61 | –0.99 | 0.56 | –1.27 | 0.28 | Unrobust |  |  |
| hsa-miR-6731-5p | 100 | 0.88 | –1.28 | 0.71 | –1.16 | 0.12 | Unrobust |  |  |
| hsa-miR-6734-5p | 99.3 | 0.79 | –1.15 | 0.82 | –1.34 | 0.19 | Unrobust |  |  |
| hsa-miR-6740-5p | 98.7 | 0.69 | –0.87 | 0.64 | –1.06 | 0.19 | Unrobust |  |  |
| hsa-miR-6741-5p | 100 | 0.89 | –0.99 | 0.69 | –0.88 | 0.11 | Unrobust |  |  |
| hsa-miR-6742-5p | 96.7 | 0.74 | –0.79 | 0.62 | –0.9 | 0.11 | Unrobust |  |  |
| hsa-miR-6743-3p | 97.7 | 0.53 | –0.45 | 0.3 | –0.4 | 0.05 | Unrobust |  |  |
| hsa-miR-6746-3p | 99 | 0.63 | –0.57 | 0.71 | –0.68 | 0.11 | Unrobust |  |  |
| hsa-miR-6748-5p | 97.7 | 0.68 | –0.82 | 0.71 | –1.07 | 0.25 | Unrobust |  |  |
| hsa-miR-6750-5p | 97.7 | 0.73 | –0.89 | 0.34 | –0.79 | 0.1 | Unrobust |  |  |
| hsa-miR-6758-5p | 98 | 0.75 | –1.07 | 0.8 | –1.21 | 0.14 | Unrobust |  |  |
| hsa-miR-6760-5p | 99.3 | 0.86 | –1.06 | 0.77 | –1.25 | 0.19 | Unrobust |  |  |
| hsa-miR-6768-5p | 100 | 0.62 | –1 | 0.82 | –1.13 | 0.13 | Unrobust |  |  |
| hsa-miR-6780a-5p | 95 | 0.71 | –0.91 | 0.71 | –1.14 | 0.23 | Unrobust |  |  |
| hsa-miR-6780b-5p | 100 | 0.92 | –1.04 | 0.93 | –1.16 | 0.12 | Unrobust |  |  |
| hsa-miR-6782-5p | 100 | 0.85 | –1.17 | 0.72 | –1.28 | 0.11 | Unrobust |  |  |
| hsa-miR-6805-3p | 100 | 0.83 | –0.83 | 0.65 | –0.95 | 0.12 | Unrobust |  |  |
| hsa-miR-6815-5p | 99 | 0.54 | –0.8 | 0.59 | –1.03 | 0.23 | Unrobust |  |  |
| hsa-miR-6822-5p | 99.3 | 0.64 | –0.95 | 0.35 | –0.84 | 0.11 | Unrobust |  |  |
| hsa-miR-6825-5p | 100 | 0.51 | –0.88 | 0.46 | –0.98 | 0.1 | Unrobust |  |  |
| hsa-miR-6833-5p | 94.3 | 0.72 | –1.04 | 0.69 | –1.27 | 0.23 | Unrobust |  |  |
| hsa-miR-6842-5p | 100 | 0.85 | –1.17 | 0.69 | –1.06 | 0.11 | Unrobust |  |  |
| hsa-miR-6849-5p | 99.7 | 0.77 | –1.37 | 0.7 | –1.14 | 0.23 | Unrobust |  |  |
| hsa-miR-6850-3p | 97.3 | 0.52 | –0.38 | 0.3 | –0.48 | 0.1 | Unrobust |  |  |
| hsa-miR-6855-5p | 99 | 0.69 | –0.96 | 0.54 | –1.17 | 0.21 | Unrobust |  |  |
| hsa-miR-6856-5p | 97.7 | 0.54 | –0.81 | 0.47 | –1.02 | 0.21 | Unrobust |  |  |
| hsa-miR-6857-5p | 98 | 0.54 | –0.73 | 0.68 | –0.92 | 0.19 | Unrobust |  |  |
| hsa-miR-6869-5p | 100 | 0.92 | –0.96 | 0 | 0 | 0.96 | Unrobust |  |  |
| hsa-miR-6870-5p | 100 | 0.92 | –1.11 | 0.92 | –1.26 | 0.15 | Unrobust |  |  |
| hsa-miR-6877-5p | 100 | 0.82 | –1.06 | 0.71 | –0.92 | 0.14 | Unrobust |  |  |
| hsa-miR-6881-5p | 96.7 | 0.74 | –0.99 | 0.78 | –1.21 | 0.22 | Unrobust |  |  |
| hsa-miR-6885-5p | 100 | 0.69 | –0.63 | 0.48 | –0.58 | 0.05 | Unrobust |  |  |
| hsa-miR-6889-5p | 100 | 0.87 | –0.85 | 0.86 | –1.01 | 0.16 | Unrobust |  |  |
| hsa-miR-6890-5p | 95 | 0.63 | –0.82 | 0.66 | –1.02 | 0.2 | Unrobust |  |  |
| hsa-miR-6895-5p | 100 | 0.56 | –1.08 | 0.61 | –0.95 | 0.13 | Unrobust |  |  |
| hsa-miR-7108-3p | 99 | 0.7 | –0.68 | 0.62 | –0.79 | 0.11 | Unrobust |  |  |
| hsa-miR-7113-3p | 98.7 | 0.73 | –0.72 | 0.79 | –0.89 | 0.17 | Unrobust |  |  |
| hsa-miR-7114-5p | 100 | 0.82 | –1.07 | 0.53 | –0.93 | 0.14 | Unrobust |  |  |
| hsa-miR-7150 | 100 | 0.93 | –1.12 | 0.94 | –1.24 | 0.12 | Unrobust |  |  |
| hsa-miR-744-5p | 100 | 0.71 | –1.09 | 0.52 | –1.2 | 0.11 | Unrobust |  |  |
| hsa-miR-766-3p | 99 | 0.51 | –0.57 | 0.47 | –0.54 | 0.03 | Unrobust |  |  |
| hsa-miR-7704 | 100 | 0.91 | –0.94 | 0.8 | –0.83 | 0.11 | Unrobust |  |  |
| hsa-miR-7843-5p | 94.3 | 0.58 | –0.65 | 0.62 | –0.98 | 0.33 | Unrobust |  |  |
| hsa-miR-7851-3p | 100 | 0.75 | –1.28 | 0.61 | –1.04 | 0.24 | Unrobust |  |  |
| hsa-miR-7854-3p | 99.7 | 0.58 | –0.8 | 0.4 | –0.95 | 0.15 | Unrobust |  |  |
| hsa-miR-8073 | 99.7 | 0.86 | –0.93 | 0.79 | –1.09 | 0.16 | Unrobust |  |  |
| hsa-miR-8078 | 99.7 | 0.54 | –1.13 | 0.41 | –1.11 | 0.02 | Unrobust |  |  |
| hsa-miR-8089 | 100 | 0.77 | –1.23 | 0.81 | –1.09 | 0.14 | Unrobust |  |  |
| hsa-miR-873-3p | 99 | 0.7 | –0.83 | 0.51 | –1 | 0.17 | Unrobust |  |  |
| hsa-miR-874-3p | 99.3 | 0.76 | –0.81 | 0.46 | –0.74 | 0.07 | Unrobust |  |  |
| hsa-miR-874-5p | 98.7 | 0.7 | –0.79 | 0.76 | –0.9 | 0.11 | Unrobust |  |  |
| hsa-miR-885-3p | 100 | 0.75 | –1.18 | 0.48 | –1.21 | 0.03 | Unrobust |  |  |
| hsa-miR-887-3p | 100 | 0.88 | –1.12 | 0.8 | –0.98 | 0.14 | Unrobust |  |  |
| hsa-miR-887-5p | 99.7 | 0.55 | –1.51 | 0.51 | –1.14 | 0.37 | Unrobust |  |  |
| hsa-miR-921 | 93 | 0.56 | –0.83 | 0.57 | –1 | 0.17 | Unrobust |  |  |
| hsa-miR-939-3p | 96.3 | 0.7 | –0.69 | 0.68 | –0.86 | 0.17 | Unrobust |  |  |
| hsa-miR-940 | 100 | 0.61 | –0.96 | 0.34 | –1.06 | 0.1 | Unrobust |  |  |
| hsa-miR-9500 | 100 | 0.67 | –1.08 | 0.45 | –0.93 | 0.15 | Unrobust |  |  |
| hsa-miR-9718 | 100 | 0.82 | –1.06 | 0.41 | –0.81 | 0.25 | Unrobust |  |  |
| hsa-miR-9901 | 99 | 0.6 | –0.64 | 0.65 | –0.78 | 0.14 | Unrobust |  |  |

Abbreviations: WGCNA, weighted gene coexpression network analysis; r, Pearson correlation coefficient.

miRNAs with underbar are 16 marker candidates.

**Supplementary Table S4. Discriminatory performance of Index-1, CA19-9, and their combination.**

| Population | Discriminant | TP | FP | FN | TN | Se | Sp | Se at 90% Sp | Sp at 90% Se | PPV | NPV | AUC  (95 % CI) | p-value |
| --- | --- | --- | --- | --- | --- | --- | --- | --- | --- | --- | --- | --- | --- |
|  |  | N |  |  |  |  |  |  |  |  |  |  |  |
| Total | CA19-9 | 168 | 14 | 59 | 236 | 0.740 | 0.944 | 0.762 | 0.403 | 0.923 | 0.800 | 0.872  (0.836-0.908) | 0.103 |
|  | Index-1 | 193 | 42 | 34 | 208 | 0.850 | 0.832 | 0.736 | 0.719 | 0.821 | 0.860 | 0.908  (0.881-0.934) |  |
|  | Combi | 215 | 54 | 12 | 196 | 0.947 | 0.784 |  |  | 0.799 | 0.942 |  |  |
| Exploratory set | CA19-9 | 93 | 9 | 41 | 141 | 0.694 | 0.940 | 0.716 | 0.285 | 0.912 | 0.775 | 0.843  (0.792-0.894) | 0.102 |
|  | Index-1 | 110 | 26 | 24 | 124 | 0.821 | 0.827 | 0.694 | 0.716 | 0.809 | 0.838 | 0.894  (0.858-0.931) |  |
|  | Combi | 124 | 34 | 10 | 116 | 0.925 | 0.773 |  |  | 0.785 | 0.921 |  |  |
| Validation set | CA19-9 | 38 | 4 | 9 | 46 | 0.809 | 0.920 | 0.830 | 0.800 | 0.905 | 0.836 | 0.937  (0.886-0.988) | 0.680 |
|  | Index-1 | 43 | 6 | 4 | 44 | 0.915 | 0.880 | 0.830 | 0.880 | 0.878 | 0.917 | 0.951  (0.907-0.996) |  |
|  | Combi | 46 | 10 | 1 | 40 | 0.979 | 0.800 |  |  | 0.821 | 0.976 |  |  |
| Independent validation set | CA19-9 | 37 | 1 | 9 | 49 | 0.804 | 0.980 | 0.826 | 0.072 | 0.974 | 0.845 | 0.873  (0.783-0.963) | 0.483 |
|  | Index-1 | 40 | 10 | 6 | 40 | 0.870 | 0.800 | 0.804 | 0.740 | 0.800 | 0.870 | 0.911  (0.849-0.972) |  |
|  | Combi | 45 | 10 | 1 | 40 | 0.978 | 0.800 |  |  | 0.818 | 0.976 |  |  |

Abbreviations: CA19-9, carbohydrate antigen 19-9; Combi, combination of Index-1 and CA19-9; TP, true positive; FP, false positive; FN, false negative; TN, true negative; Se, sensitivity; Sp, specificity; Se at 90% Sp, sensitivity at 90% of specificity; Sp at 90% Se, specificity at 90% of sensitivity; PPV, positive predictive value; NPV, negative predictive value; AUC, area under the ROC, CI, confidence interval.

**Supplementary Table S5.** **Discriminatory performance of Index-1 when changing the threshold for CA19-9 levels.**

|  | Test vs. Control | CA19-9 (U/mL) | | | |
| --- | --- | --- | --- | --- | --- |
|  |  | ≥37 | <37 | <15 | Any |
| Sensitivity | PBca vs. healthy  (n) | 0.869 (168 vs. 14) | 0.797 (59 vs. 236) | 0.727 (33 vs. 186) | 0.850 (227 vs. 250) |
|  | Pancreatic cancer vs. healthy  (n) | 0.891 (129 vs. 14) | 0.825 (40 vs. 236) | 0.696 (23 vs. 186) | 0.876 (169 vs. 250) |
| Specificity |  | 0.857 | 0.831 | 0.839 | 0.832 |

Abbreviations: CA19-9, carbohydrate antigen 19-9; PBca, pancreatobiliary cancer

**Supplementary Table S6.** **miRNA expression in pancreatic cancer lines and miR-665 effects on cell proliferation.**

|  | Average | SD | p-value |
| --- | --- | --- | --- |
| Control | 3275603 | 112252 |  |
| miR-1343-5p | 3071968 | 309620 | 0.345 |
| Control | 2691800 | 48508 |  |
| miR-4632-5p | 2712551 | 153644 | 0.834 |
| Control | 3275603 | 112252 |  |
| miR-4665-5p | 3003413 | 44615 | 0.018 |
| Control | 3275603 | 112252 |  |
| miR-665 | 1831884 | 269121 | 0.001 |
| Control | 2691800 | 48508 |  |
| miR-6803-5p | 2790509 | 53590 | 0.077 |

n = 3 for each setting. p-values were calculated using Student’s t-test.

Abbreviations: SD, standard deviation
